# Phylogenetic analysis of a gene cluster encoding an additional, rhizobial-like type III secretion system that is narrowly distributed among *Pseudomonas syringae* strains

**DOI:** 10.1186/1471-2180-12-188

**Published:** 2012-09-02

**Authors:** Anastasia D Gazi, Panagiotis F Sarris, Vasiliki E Fadouloglou, Spyridoula N Charova, Nikolaos Mathioudakis, Nicholas J Panopoulos, Michael Kokkinidis

**Affiliations:** 1 Department of Biology, University of Crete, Vasilika Vouton, P.O. Box 2208, Heraklion, Crete GR 71409, Greece; 2 Institute of Molecular Biology & Biotechnology, Foundation of Research & Technology, Vasilika Vouton, Heraklion, Crete GR 71110, Greece; 3 Institute Pasteur, 28 rue du Docteur Roux, Paris, 75015, France; 4 School of Medicine, University of Crete, Vasilika Vouton, Heraklion, Crete, GR 71409, Greece; 5 Department of Molecular Biology and Genetics, Democritus University of Thrace, Alexandroupolis, GR 68100, Greece

**Keywords:** *Hrc1*Type III secretion system, *Pseudomonas syringae*, *Rhizobium* Type III secretion system, Phylogenetic analysis, Pathogenicity, Gene organization, Horizontal transfer events, Common ancestry, Evolutionary relationships, RT-PCR

## Abstract

**Background:**

The central role of Type III secretion systems (T3SS) in bacteria-plant interactions is well established, yet unexpected findings are being uncovered through bacterial genome sequencing. Some *Pseudomonas syringae* strains possess an uncharacterized cluster of genes encoding putative components of a second T3SS (T3SS-2) in addition to the well characterized *Hrc1* T3SS which is associated with disease lesions in host plants and with the triggering of hypersensitive response in non-host plants. The aim of this study is to perform an *in silico* analysis of T3SS-2, and to compare it with other known T3SSs.

**Results:**

Based on phylogenetic analysis and gene organization comparisons, the T3SS-2 cluster of the *P. syringae* pv. phaseolicola strain is grouped with a second T3SS found in the pNGR234b plasmid of *Rhizobium* sp. These additional T3SS gene clusters define a subgroup within the *Rhizobium* T3SS family. Although, T3SS-2 is not distributed as widely as the *Hrc1* T3SS in *P. syringae* strains, it was found to be constitutively expressed in *P. syringae* pv phaseolicola through RT-PCR experiments.

**Conclusions:**

The relatedness of the *P. syringae* T3SS-2 to a second T3SS from the pNGR234b plasmid of *Rhizobium* sp., member of subgroup II of the rhizobial T3SS family, indicates common ancestry and/or possible horizontal transfer events between these species. Functional analysis and genome sequencing of more rhizobia and *P. syringae* pathovars may shed light into why these bacteria maintain a second T3SS gene cluster in their genome.

## Background

Gram-negative proteobacteria deploy various types of protein secretion systems for exporting selected sets of proteins to the cell surface, the extracellular space or into host cells [1,2]. Type III Secretion Systems (T3SS) are directly related to pathogenicity or to symbiosis with higher organisms and constitute essential mediators of the interactions between gram-negative bacterial cells and eukaryotic ones [3-8] as the T3SS efficiently translocates bacterial proteins (effectors) directly into the host cell cytoplasm when fully developed.

The T3SS apparatus comprises three distinct parts: a) the basal body, which forms a cylindrical base that penetrates the two bacterial membranes and the periplasmic space; b) the extracellular part with the needle or the pilus as its main feature which is formed through the polymerization of specialized protein subunits that are T3SS substrates themselves; and c) the cytoplasmic part, which forms the export gate for secretion control. This apparatus is built by specific core proteins encoded by a conserved subset of genes tightly organized in gene clusters with counterparts in the bacterial flagellum [6,7].

Phylogenetic analyses of the core T3SS proteins revealed that the T3S systems evolved into seven distinct families that spread between bacteria by horizontal gene transfer. (1) The *Ysc*-T3SS family, named after the archetypal *Yersinia* system, is present in α-, β-, γ-, and δ- proteobacteria. At least in α-proteobacteria the system confers resistance to phagocytosis and triggers macrophage apoptosis. (2) The *Ssa-Esc*-T3SS family is named after the archetypal T3SS of enteropathogenic and enterohemorrhagic *E.coli*. (3) The *Inv-Mxi-Spa*-T3SS family named after the Inv-Spa system of *Salmonella enterica* and the Inv-Mxi T3S system of *Shigella* spp. The family members trigger bacterial uptake by nonphagocytic cells.(4) The *Hrc-Hrp1*- and (5) the *Hrc-Hrp2*-T3SS families are present in plant pathogenic bacteria of the genus *Pseudomonas, Erwinia, Ralstonia* and *Xanthomonas*. The two families are differentiated on the basis of their genetic loci organization and regulatory systems. (6) The *Rhizobiales*-T3SS family (hereafter referred to as *Rhc*-T3SS) is dedicated to the intimate endosymbiosis serving nitrogen fixation in the roots of leguminous plants. (7) Finally the *Chlamydiales*-T3SS is present only in these strictly intracellular nonproteobacteria pathogens [8,9]. The phylogenetic trees obtained by the above analysis were totally incongruent with the evolutionary tree of bacteria based on 16S rRNA sequences. These results imply that T3S systems did not originate within their present host bacteria, but spread through horizontal gene transfer events [9]. Furthermore, apart from a high degree of gene homologies within the T3SS families, the overall genetic organization (synteny) is also conserved [8].

In this study, we present a detailed phylogenetic and gene synteny analysis of core T3SS proteins. This analysis reveals the presence of three distinct *Rhc*-T3SS family subgroups. From these subgroups, the one designated as subgroup II was found to comprise T3S systems from various *Pseudomonas syringae* strains as well as from *Rhizobium* sp. NGR234. The T3SS of subgroup II will be hereafter referred to as T3SS-2, because these systems exist in their bacterial hosts next to the well-studied T3SS from the pNGR234a plasmid of *Rhizobium* sp. and the *Hrc1-Hrp1* T3S system of *P. syringae*. Interestingly, at least two of the genes from the additional T3SS-2 gene cluster in *P. syringae* pv phaseolicola strain 1448a were found to be transcriptionally active.

## Methods

### Sequence analysis

#### Genomic regions

The regions comprising and surrounding the T3SS-2 gene clusters of *P. syringae* pv phaseolicola 1448a, *P. syringae* pv oryzae str. 1_6, *P. syringae* pv tabaci ATCC11528, *Rhizobium* spp. NGR234 and the regions comprising and surrounding the unique T3SS gene clusters of *Bradyrhizobium japonicum* USDA 110, *Rhizobium etli* CIAT 652 and *R. etli* CNF 42 were retrieved from the NCBI Genome database. In the cases of *P. syringae* pv tabaci ATCC11528 and *P. syringae* pv aesculi the nucleotide sequence in the region close to the T3SS gene cluster was retrieved (GenBank: N° ACHU01000133 and N° ACXS01000083.1 respectively) after being identified through MegaBLAST searches and found to be present in *P. syringae* pv phaseolicola 1448a, but absent from *P. syringae* pv tomato DC3000 and *Pseudomonas syringae* pv syringae B728A; coding sequences were identified with NCBI’s ORF Finder tool.

#### Amino acid sequence analysis

Each coding sequence annotated in the T3SS gene clusters of *P. syringae* pv phaseolicola 1448a, *R. etli* CIAT 652 and *Rhizobium* spp. NGR234 was analyzed by Psi-BLAST searches [10] against the NCBI non-redundant database reduced for bacteria using the following parameters: BLOSUM 65 substitution matrix; expected threshold 10; word size 3; gap costs: existence: 11, extension 1; the filter for low complexity regions was set to on. The number of descriptions and alignments to be reported was set to 500 and conditional compositional adjustments were on. The program FoldIndex© was used with default parameters for the prediction of structural disorder propensity from the amino acid sequences [11]. Secondary structure predictions were performed with PSIPRED [12]. Physical and chemical parameters of sequences under study were estimated by ProtParam [13]. Coiled coil predictions and assignment of the heptad repeat positions in proteins were produced in COILS [14] and MATCHER [15] respectively. Sequence threading techniques and fold-recognition algorithms were used to identify distant homologs. 3-D structural profiles for T3SS proteins were predicted from sequence data was performed using the PHYRE pipeline [16]. The program Memstat3 [17] was used for the prediction of membrane α-helices in proteins.

#### Nucleotide sequence analysis

The gene synteny of the T3SS-2 clusters of *P. syringae* pv phaseolicola 1448a, *P. syringae* pv oryzae str. 1_6, *P. syringae* pv tabaci ATCC11528, *Rhizobium* spp. NGR234 and the gene synteny of the unique T3SS gene clusters of *B. japonicum* USDA 110, *R. etli* CIAT 652, *R. etli* CNF 42, were compared to other known T3SS gene clusters of various bacteria using the BLASTN and BLASTP tools of the Genbank. Codon Usage Bias analysis was performed using DnaSP v5 [18].

### Phylogenetic analysis

T3SS core protein sequences were retrieved using Psi-BLAST searches with the *P. syringae* pv phaseolicola 1448a T3SS-2 gene cluster coding frames and were aligned with the multiple alignment method ClustalW, version 1.8 [19].

Phylogenetic relations were inferred using the neighbour-joining method [20] implemented in the MEGA4 software [21]. The bootstrap consensus tree inferred from 1000 replicates [22] is taken to represent the evolutionary history of the amino acid sequences analyzed [22]. Branches corresponding to partitions reproduced in less than 50% bootstrap replicates are collapsed. The percentage of replicate trees in which the associated taxa clustered together in the bootstrap test (1000 replicates) are shown next to the branches [22]. The tree is drawn to scale, with branch lengths in the same units as those of the evolutionary distances used to infer the phylogenetic tree. The evolutionary distances were computed using the Poisson correction method [23] and are in the units of the number of amino acid substitutions per site. All positions containing alignment gaps and missing data were eliminated only in pair wise sequence comparisons.

### Cultivation

*P. syringae* strains were routinely grown at 28°C in LB medium. Bacteria of overnight culture were collected at an OD (optical density) of 0.8. The bacterial pellet was washed with 10 mM MgCl_2_ and the cells were resuspended (OD: 0.6-0.7) in Hrp-induction media [24] for overnight cultivation at 28°C. The next day the bacterial cells were collected (OD: 0.7-0.8) for RNA extraction.

### RT-PCR

For the RT-PCR reactions, total RNA was extracted from overnight bacterial cultures of *P. syringae* pv phaseolicola 1448a and *P. syringae* pv tomato DC3000, using both LB and Hrp-induction media [24]. Total RNA was treated with RNase-free DNase I for 45 min at 37°C [25]. From both culture conditions equal amounts of the extracted total RNA were subjected to RT-PCR with gene specific primers for the PSPPH_2530, PSPPH_2524 and 16S rDNA genes, using the OneStep RT-PCR kit according to the manufacturer’s instructions (QIAGEN). For negative control, PCR was performed on the total RNA without Reverse Transcriptase assay, using the 16S rDNA primers, in order to accredit no DNA contamination in the total RNA isolation samples. The RT-PCR products were then analyzed by agarose gel electrophoresis. Primers sequences for 16S RNA were 5^′^-CGGGTACTTGTACCTGGTGGC-3^′^ and 5^′^-CTTGCCAGTTTTGGATGCAGTTC-3^′^, for PSPPH_2530 were 5^′^-AGGCCCTGACGACGCTGCTG-3^′^ and 5^′^-CCAGGTGCCTGTGTTCGGCAGT-3^′^ and for PSPPH_2524 5^′^-TCCTGCTGTGCCTGTTATCCGGCG-3^′^ and 5^′^-GACGGTCGGTAGCGACTTGAGTGAC-3^′^.

## Results and discussion

### Analysis of core components of *P. syringae* T3SS-2 and the *Rhc*-T3SS family

#### Phylogenetic analysis of core proteins

In the subsequent sections the unified nomenclature for T3SS proteins (Table 1) will be followed [26]. The phylogenetic analysis of various T3SS core proteins (including T3SS-2 proteins), e.g. SctU (RhcU/HrcU/YscU/FlhB and their homologues), SctV (RhcV/HrcV/LcrD/FlhA homolog proteins), SctQ (RhcQ/HrcQ/YscQ/FliN/ and their homologues) and the T3SS ATPases SctN (RhcN/HrcN/YscN/FliI and homologues), confirmed the broad classification of the non-flagellar T3SS into seven families. However, the T3SS-2 proteins were grouped in the same clade with the *Rhc* T3SS proteins with high bootstrap values, suggesting that these lineages share a more recent common origin than with other T3SS families.

**Table 1:** **T3SS proteins assigned under the unified nomenclature using the *Sct *(SeCreTion) prefix**

**T3SS family**	***Unified nomenclature vs Species***	**SctV**	**SctW**	**SctN**	**SctO**	**SctP**	**SctQ**	**SctR**	**SctS**	**SctT**	**SctU**	**SctC**	**SctD**	**SctF**	**SctI**	**SctJ**	**SctK**	**SctL**
***Ysc***	*Yersinia* sp.	LcrD	YopN	YscN	YscO	YscP	YscQ	YscR	YscS	YscT	YscU	YscC	YscD	YscF	YscI	YscJ	YscK	YscL
	*Pseudomonas aeruginosa*	PcrD	PopN	PscN	PscO	PscP	PscQ	PscR	PscS	PscT	PscU	PscC	PscD	PscF	PscI	PscJ	PscK	PscL
***Inv-Mxi-Spa***	*Shigella flexneri*	MxiA	Orf15MxiC	SpaL Spa47	SpaM Spa13	SpaN Spa32	SpaO Spa33	SpaP Spa24	SpaQ Spa9	SpaR Spa29	SpaU Spa40	MxiD	MxiG	MxiH	MxiI	MxiJ	MxiK	MxiN
	*Salmonella enterica*	InvA	InvE	InvC	InvI	InvJ	SpaO InvK	SpaP InvL	SpaQ	SpaR InvN	SpaS	InvG	PrgH	PrgI	PrgJ	PrgK	OrgA	OrgB
***Ssa-Esc***	*Salmonella enterica*	SsaV		SsaN	SsaO	SsaP	SsaQ	SsaR	SsaS	SsaT	SsaU	SpiA SsaC	SpiB SsaD	SsaG		SsaJ		SsaK
	EPEC	SepA	SepL	SepB	Orf15		SepQ	EscR	EscS	EscT	EscU	SepC		ORFD2	rOrf8	SepD		ORF5
***Chlamydiales***	*Chlamydia trachomatis*	CdsV	CopN	CdsN	CT670 CdsO	CT671 CdsP	CdsQ	CdsR	CdsS	CdsT	CdsU	CdsC	CdsD	CsdF		CdsJ		CdsL
***Hrp-Hrc1***	*Pseudomonas syringae*	HrcV	HrpJ	HrcN	HrpO	HrpP	HrcQ_A_ & HrcQ_B_	HrcR	HrcS	HrcT	HrcU	HrcC	HrpQ	HrpA	HrpB	HrcJ	HrpD	HrpE
	*Erwinia amylovora*	HrcV	HrpJ	HrcN	HrpO	HrpP	HrcQ	HrcR	HrcS	HrcT	HrcU	HrcC	HrpQ	HrpA	HrpB	HrcJ		HrpE
***Hrp-Hrc2***	*Burkcholderia pseudomallei*	SctV		SctN	HrpD	HpaC	SctQ	SctR	SctS	SctT	SctU	SctC	SctD			SctJ		SctL
	*Ralstonia solanacearum*	HrcV		HrcN	HrpD	HpaP	HrcQ	HrcR	HrcS	HrcT	HrcU	HrcC	HrpW	HrpY	HrpJ	HrcJ		HrpF
	*Xanthomonas campestris*	HrcV	HpaA	HrcN	HrpB7	HpaP HpaC	HrcQ	HrcR	HrcS	HrcT	HrcU	HrcC	HrpD5	HrpE	HrpB2	HrcJ		HrcL HrpB5
***Rhc***	*Subgroup I Rhizobium* pNGR234a	Y4yR	-	RhcN	Y4yJ	-	RhcQ	RhcR	RhcS	RhcT	RhcU	NolW RhcC_1_ & RhcC_2_	Y4yQ		NolU	NolT		NolV
	*Subgroup II P. syringae*	Hrc_*II*_V		Hrc_*II*_N	Hrc_*II*_O		Hrc_*II*_Q	Hrc_*II*_R	Hrc_*II*_S	Hrc_*II*_T	Hrc_*II*_U	Hrc_*II*_C_1_ Hrc_*II*_C_2_	Hrp_*II*_Q			Hrc_*II*_J		Hrp_*II*_E
	*Subgroup II Rhizobium* pNGR234b	Rhc_*II*_V	-		Rhc_*II*_O	-	Rhc_*II*_Q	Rhc_*II*_R	Rhc_*II*_S	Rhc_*II*_T	Rhc_*II*_U	Rhc_*II*_C_1_ & Rhc_*II*_C_2_	Rhp_*II*_Q					Rhc_*II*_L
	*Subgroup III Rhizobium etli*	RhcV	-	RhcN	RhcO	-	RhcQ	RhcR	RhcS	RhcT	RhcU	RhcC_1_			NolU	RhcJ		RhcL
**Flagellar**		FlhA		FliI	FliJ		FliY FliM & FliN	FliP	FliQ	FliR	FlhB		FliG			FliF		FliH

Shaded boxes are indicative of proteins with analog function but no sequence homology to the *Ysc* T3SS family. Double names are also reported for various cases.

**Figure 1:**
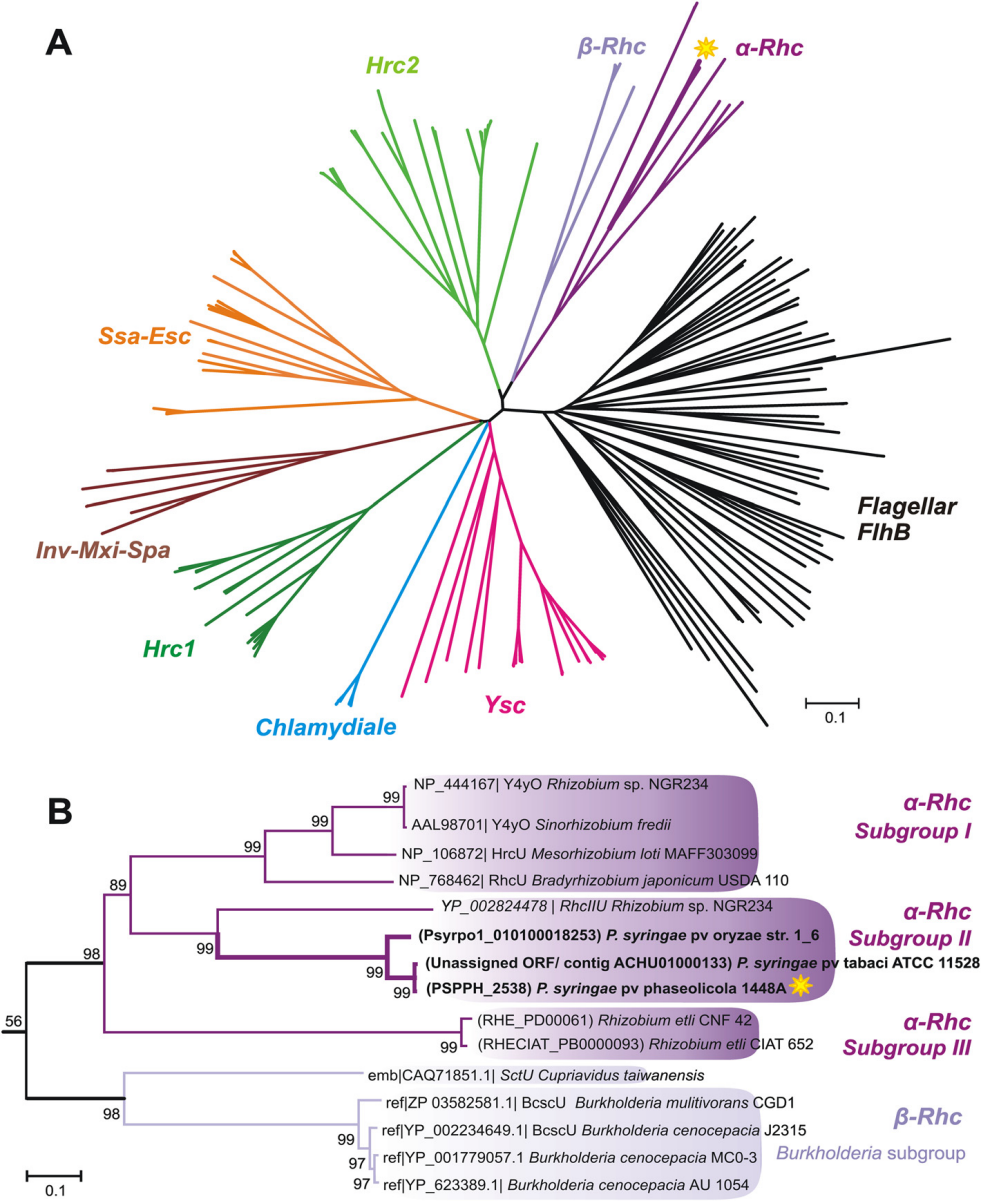
**Evolutionary relationships of SctU proteins.** The yellow star indicates the position of the *P. syringae* pv phaseolicola 1448a Hrc_*II*_U. **A**. The phylogram of 192 SctU sequences with the eight main families named according to Troisfontaines & Cornelis (2005) [8], while the flagellum proteins are depicted in black. The T3SS family encompasing the *β-rhizobium Cupriavidus taiwanensis* and of *Burkholderia cenocepacia* group is indicated here with a light purple color (marked as β-Rhc). Branches corresponding to partitions reproduced in less than 50% bootstrap replicates are collapsed. There were a total of 686 positions in the final dataset. Phylogenetic analyses were conducted in MEGA4 [21]. **B**. The *Rhc* T3SS clade as derived from the phylogram in A, groups the *P. syringae* Hrc_*II*_U sequences close to the Rhc_*II*_U protein of the *Rhizobium* sp. NGR234 T3SS-2. The values at the nodes are the bootstrap percentages out of 1000 replicates. The locus numbers or the protein accession number of each sequence is indicated.

**Figure 2:**
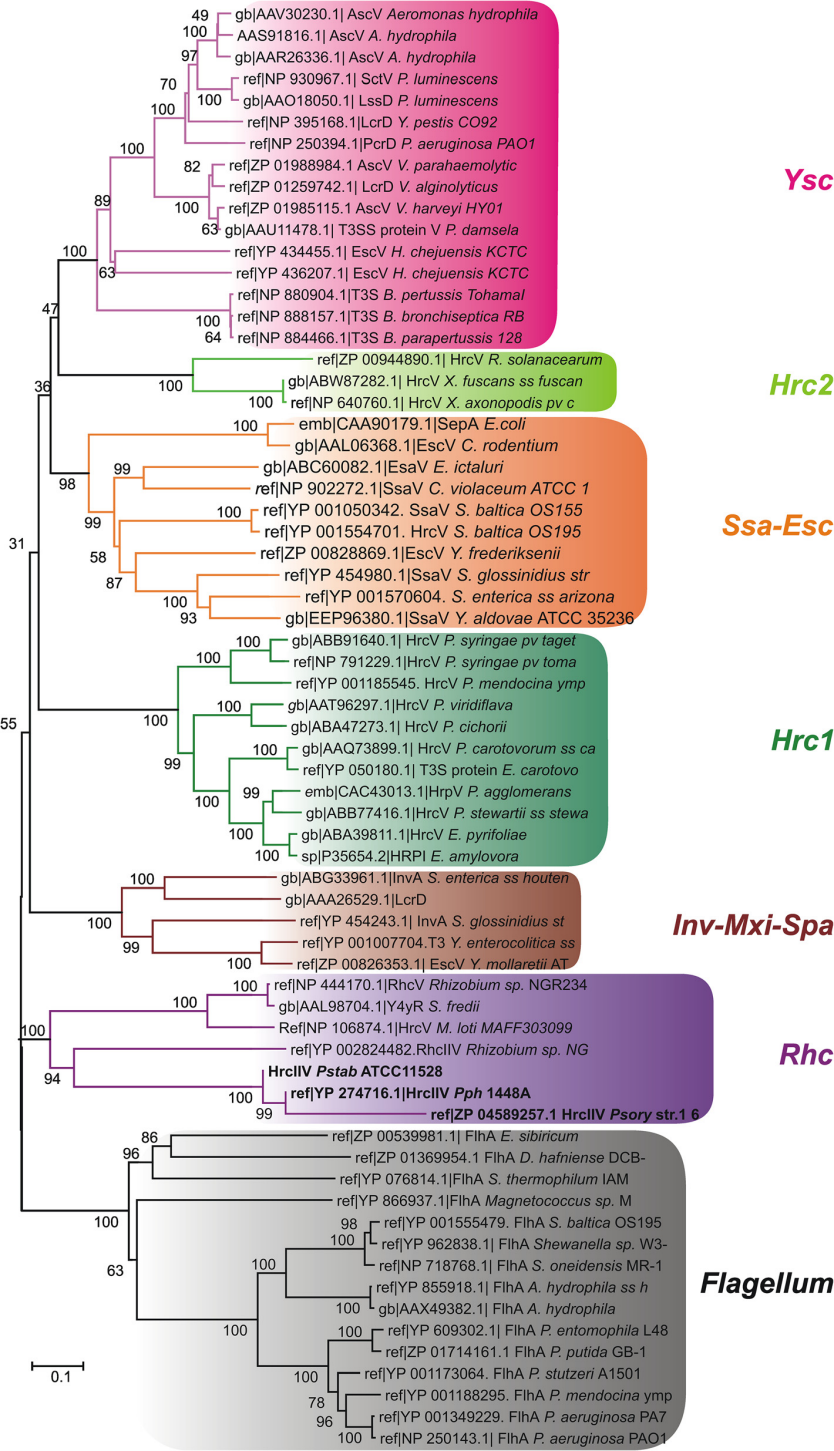
**Evolutionary relationships of SctV proteins.** Classification of the SctV T3SS proteins into the main T3SS/flagellar families. The colouring scheme of Figure 1 is used.

Interestingly, the *Rhc* T3SS family can be further subdivided into three subgroups: Subgroup I is represented by the well-known T3SSs of *Rhizobium* sp. NGR234, and *B. japonicum* USDA 110 while subgroup III is represented by the T3SS present in *R. etli.* Proteins from the T3SS-2 system of various *P. syringae* strains are grouped closer to the T3SS-2 of *Rhizobium* sp. NGR234 (Figure 1, 2, Additional files 1, Additional file 2 & Additional file 3: Figures S1, S2 & S3), forming the subgroup II of the *Rhc* T3SS family.

#### All required core T3SS components are present in the T3SS- of P. syringae strains

BLASTP and Psi-BLAST searches revealed the main T3SS components of the novel T3SS-2 gene cluster of *P. syringae* pv phaseolicola 1448a which are also conserved in *P. syringae* pv oryzae str. 1_6, *P. syringae* pv tabaci ATCC11528 (Additional file 4: Table S1) and *P. syringae* pv aesculi. Similar searches and comparisons were also carried out with the T3SSs of *R. etli* CNF 42, *R. etli* CIAT 652 and *Rhizobium* sp. strain NGR234. In the following, the prefix Hrc_*II*_ will be used to specify the conserved T3SS-2 proteins of *P. syringae* pv phaseolicola 1448a, *P. syringae* pv oryzae str. 1_6 and *P. syringae* pv tabaci, while the prefix Rhc_*II*_ will be used to distinguish the *Rhc* proteins of the T3SS-2 gene cluster found in plasmid pNGR234b of *Rhizobium* sp. NGR234 (see below). The T3SS protein nomenclature when used is indicated by the prefix *Sct* according to Table 1.

All major T3SS core proteins were found in the T3SS gene clusters mentioned above, including the T3SS ATPase protein SctN (RhcN/HrcN/YscN/FliI homolog), its negative regulator SctL (NolV/HrpE/YscL/FliH homolog), the two T3SS gate proteins SctU and SctV (RhcU/HrcU/YscU/FlhB and RhcV/HrcV/LcrD/FlhA homologs respectively), the protein building the inner ring of the T3SS basal body SctJ (RhcJ/HrcJ/YscJ homolog), the protein building the cytoplasmic ring SctQ (RhcQ/HrcQ/YscQ/FliY homolog) and the three core membrane proteins SctR, SctS, SctT (RhcRST/HrcRST/YscRST/FliPQR homologs) (Additional file 4: Table S1).

**Figure 3:**
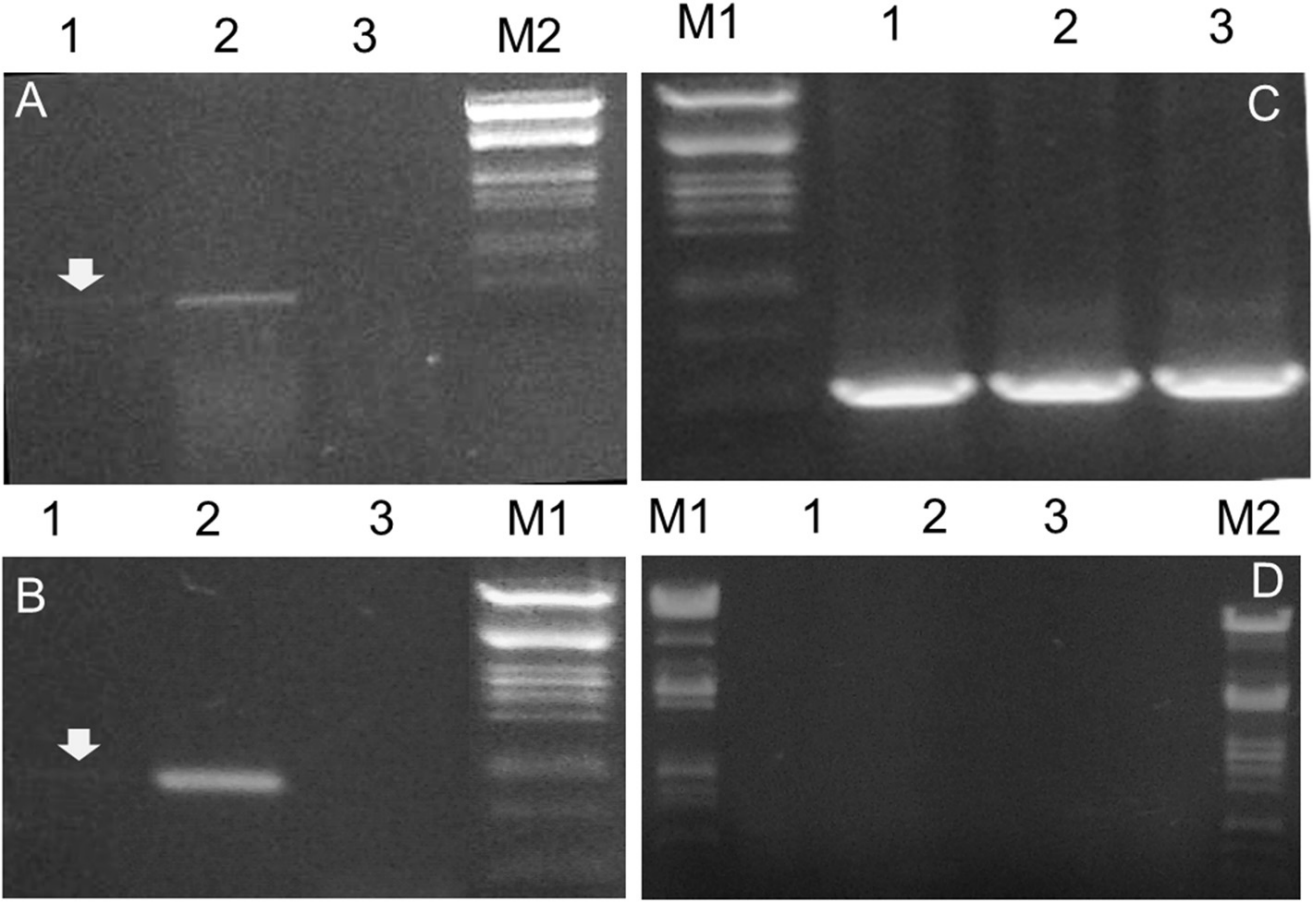
**RT-PCR analysis for the PSPPH_2530, PSPPH_2524 and 16S gene expression in bacterial total RNA. A.** RT-PCR analysis for the PSPPH_2524 expression: **1)** on total RNA from *P. syringae* pv phaseolicola 1448a cultivated in Hrp-induction medium, **2)** on total RNA from *P. syringae* pv phaseolicola 1448a cultivated in LB medium, **3)** on total RNA from *P. syringae* pv tomato DC3000 cultivated in LB medium (as a negative control). **B.** RT-PCR analysis for the PSPPH_2530 expression: **1)** on total RNA from *P. syringae* pv phaseolicola 1448a cultivated in Hrp-induction medium, **2)** on total RNA from *P. syringae* pv phaseolicola 1448a cultivated in LB medium, **3)** on total RNA from *P. syringae* pv tomato DC3000 cultivated in LB medium (as a negative control). **C.** RT-PCR analysis for the 16S rDNA expression (as a positive control): **1)** on total RNA from *P. syringae* pv phaseolicola 1448a cultivated in Hrp-induction medium, **2)** on total RNA from *P. syringae* pv phaseolicola 1448a cultivated in LB medium, **3)** on total RNA from *P. syringae* pv tomato DC3000 cultivated in LB medium. **D.** Negative control PCR was performed on the total RNA isolates from **1)***P. syringae* pv phaseolicola 1448a cultivated in Hrp-induction medium **2)***P. syringae* pv phaseolicola 1448a cultivated in LB medium, **3)***P. syringae* pv tomato DC3000 cultivated in LB medium, without Reverse Transcriptase assay using the 16S rDNA primers in order to accredit no DNA contamination in the total RNA samples. PCR products were electrophoretically resolved on ethidium bromide (0.5 μg mL^-1^)-containing agarose gels (1.5%, w/v). M1: λ DNA digested with *PstI*, M2: λ DNA digested with *EcoRI-HindIII*. Even though the total mRNA templates were equal for all PCR samples, the signals in hrp induction medium are very weak, so they have been highlighted by an arrow.

It is noteworthy that the promoter regions of the T3SS-2 ORFs/operons of *P. syringae* pv phaseolicola 1448a, do not appear to harbor "hrp box" elements like those which have been described for the T3SS-1 genes of various *P. syringae* strains [27]. This, coupled with the low expression level seen in minimal media (Figure 3), leave open the question whether T3SS-2 in this or other *P. syringae* strains is expressed under *in planta* conditions and whether it is plays a role in their phytopathogenic potential or in any other aspect of their life cycle.

#### The split secretin gene

**Figure 4:**
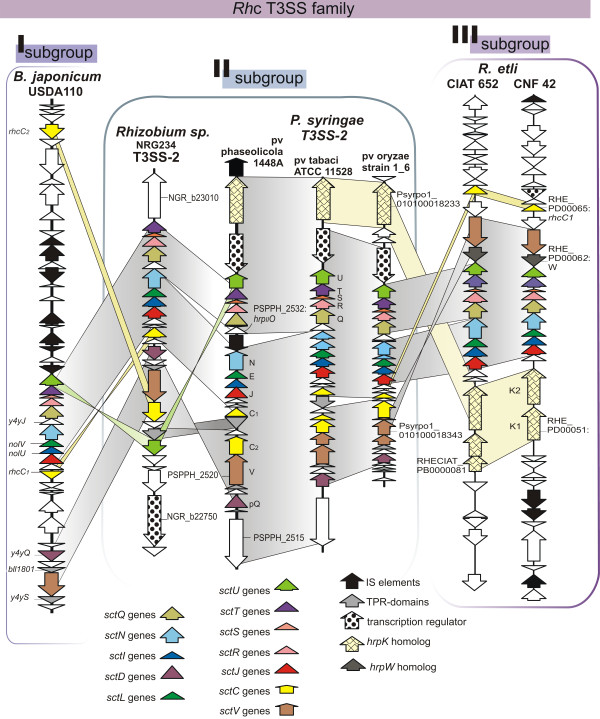
**Genetic organization of the *Rhc *T3SS gene clusters, indicating the diversification of three main subgroups.** ORFs are represented by arrows. White arrows indicate either low sequence similarities between syntenic ORFs like the PSPPH_2532: *hrpO*_*II*_ case or ORFs not directly related to the T3SS gene clusters that were excluded from the study. Homologous ORFs are indicated by similar coloring or shading pattern. Only a few loci numbers are marked for reference. Gene symbols (N, E, J etc.) for the T3SS-2 genes are following the *Hrc1* nomenclature. 1) Subgroup I cluster (*Rhc-I),* is represented by *Bradyrizhobium japonicum* USDA110 and includes also the T3SS present on the pNGR234a plasmid of strain NGR234 (not shown); 2) Subgroup II (*Hrc*_*II*_*/Rhc*_*II*_), represented by the T3SS-II gene clusters of *Rhizobium* sp. NGR234 pNGR234b plasmid [38] , *P. syringae* pv phaseolicola *1448A*[44], *P. syringae* pv tabaci ATCC 11528 and *P. syringae pv oryzae* str. 1_6 (this study, see Materials and Methods); and 3) subgroup III, represented by the sole T3SS of the *Rhizobium etli* CIAT652 (plasmid b) and the *R. etli* CNF42 plasmid d [37]. Gene products of the *Hrc*_*II*_*/Rhc*_*II*_ supgroup II T3SS share greater sequence homologies with each other than with genes of subgroups I and III (Additional file 4: Table S1).

A distinguishing feature of gene organization in *Rhc* T3SS clusters is a split gene coding for the outer membrane secretin protein SctC, i.e. a HrcC/YscC homologue [28]. This is also true for the subgroup II *Rhc* T3SS gene clusters. In the T3SS-2 clusters of the three *P. syringae* pathovars the secretin gene is split in two ORFs (Figure 4, Additional file 4: Table S1). In *P. syringae* pv phaseolicola *1448a*, loci PSPPH_2524 (*hrc*_*II*_*C1*) and PSPPH_2521 (*hrc*_*II*_*C2*) code for the N-terminal and the C-terminal part of secretin, respectively, of a HrcC/YscC homolog. Comparisons of Hrc_*II*_C1 and Hrc_*II*_C2 with the RhcC1 and Rhc2 proteins of *Rhizobium* sp. NGR234 are given in Additional file 5: Figure S4, respectively. A similar situation occurs in *P. syringae* pv oryzae str. 1_6 while in *P. syringae* pv tabaci ATCC11528 *hrc*_*II*_*C2* gene is further split into two parts. However in *P. syringae* pv phaseolicola 1448a and *P. syringae* pv tabaci ATCC11528 the two *hrc*_*II*_*C1, hrc*_*II*_*C2* genes are only separated by an opposite facing ORF coding for a TPR-protein, while in the subgroup I *Rhc* T3SS these two genes are separated even further (Figure 4). Although the functional significance of the split secretin gene is not known, there are reports of constitutive expression of the *rhcC1* gene in contrast to the rest of the T3SS operons in rhizobia [29,30]. In subgroup III only the *rhcC1* could be identified (RHECIAT_PB0000097 in the *R. etli* CIAT 652 and RHE_PD00065 in *R. etli* CNF 42) in Psi-BLAST searches using the Hrc_*ΙΙ*_C1 protein sequence as query (25% identity to RhcC1 of *Rhizobium* sp. NGR234) (Figure 4).

#### The Hrc_II_Q protein

The PSPPH_2534 locus (designated *hrc*_*II*_*Q*) in the T3SS-2 cluster of *P. syringae* pv phaseolicola 1448A codes for a polypeptide chain of 301 residues, which has sequence similarities with members of the HrcQ/YscQ/FliY family. Members of this family usually consist of two autonomous regions [26] which either are organized as two domains of a single protein or can be split up into two polypeptide chains. The Hrc_*II*_Q is comparable in length with the long proteins of the family. The same is true in the *Rhc*-T3SS case, where an HrcQ ortholog is found. In agreement with the other HrcQ/YscQ/FliY members the sequence conservation is especially high at the C-terminus [31,32]. In the originally described T3SS-1 (*Hrc-Hrp1*) of *P. syringae* strains this gene is split into two adjacent ORFs coding for separate polypeptides (HrcQ_A_ and HrcQ_B_). No splitting occurs however in the T3SS-2 clusters of the *P. syringae* strains.

#### The HrpO-like protein

A conserved feature in gene organization of T3SS gene clusters and the flagellum is the presence of a small ORF downstream of the gene coding for the ATPase (*hrcN/yscN/fliI* homologue). These ORFs code for proteins of the HrpO/YscO/FliJ family, a diverse group characterized by low sequence similarity, and heptad repeat motifs suggesting a high tendency for coiled-coil formation and a propensity for structural disorder [33]. Such a gene is also present in the *Rhizobium* NGR234 T3SS-2 but is absent from the subgroup III *Rhc-*T3SS where the *rhcQ* gene is immediately downstream of the *rhcN* gene (Figure 4). In the *P. syringae* pathovars included in Figure 4 there is a small ORF (PSPPH_2532 in strain *P. syringae* pv phaseolicola 1448A, Figure 4) coding for a polypeptide wrongly annotated as Myosin heavy chain B (MHC B) in the NCBI protein database. Sequence analysis of this protein and its homologs in the other two *P. syringae* strains using BLASTP searches did not reveal any significant similarities to other proteins. However, these small proteins are predicted as unfolded in their entire length, while heptad repeat patterns are recognizable in the largest part of their sequence, thus strongly resembling the properties of members of the HrpO/YscO/FliJ family [33], (Additional file 6: Figure S5). A potentially important feature in the *P. syringae* pv phaseolicola 1448a T3SS-2 cluster is a predicted transposase gene between the ORF coding for the above described HrpO/YscO/FliJ family member and the ORF for the Hrc_II_N ATPase (Figure 4); this gene is absent from the *P. syringae* pv tabaci and *P. syringae* pv oryzae str.1_6 T3SS-2 clusters. The insertion of the transposase gene does not disrupt genes *hrc*_*II*_*N* or *hrp*_*II*_*O* as concluded by amino acid sequence comparison with other members of the SctN and SctO protein families respectively (including ORFs from other T3SS-2 *P. syringae* strains). These genes are capable of producing the respective full-length proteins and no premature termination, due to transposase insertion, is observed.

#### The HrpQ-like protein

Another common feature of *P. syringae* T3SS-2 and the *Rhizobium* T3SSs excluding subgroup III, is a gene usually positioned upstream of the *sctV* gene (*rhcV/hrcV/lcrD/flhA* homolog) and in close proximity to it. Psi-BLAST searches for the PSPPH_2517 encoded protein revealed moderate similarities to the HrpQ/YscD family of T3SS proteins; these were confirmed by sequence threading techniques. For example, a segment of of PSPPH_2517 corresponding to 45% of its amino acid sequence scores an E-value of 2e-05 and a 26% identity with YscD protein from *Yersinia enterocolitica* (ref|YP_006007912.1); the same segment scores an E-value of 1e-13 with 25% identity to the 90% of its sequence with the equivalent protein from *B. japonicum* USDA110 (ref| NP_768443.1). The chosen folding templates belong to various forkhead - associated (FHA) protein domains from different origins. FHA cytoplasmic domains characterize the YscD/EscD protein family and may suggest phosphopeptide recognition interactions [34]. A protein with the above characteristics is present in the *B. japonicum* USDA110 T3SS cluster (encoded by the *y4yQ* gene) while an ortholog could not be identified in the *R. etli* T3SS.

### Gene clusters organization in the *Rhc-*T3SS family and the *P. syringae* T3SS-2

Subgroup I of the *Rhc*-T3SS family comprises the first described and well characterized T3SS-1 of *Rhizobium* NGR234 present in the plasmid pNGR234a [35], along with that of *B. japonicum* USDA110 and others [36]. The T3SS core genes in this case are organized in three segments. The biggest segment harbors the genes *rhcU*, *rhcT, rhcS, rhcR, rhcQ, y4yJ, rhcN, nolV, nolU, rhcJ, nolB,* in the same DNA strand with the *rhcC1* gene flanking the *nolB* gene in the opposite strand (Figure 4, Subgroup I). The second one harbors the *rhcV* gene usually between the *y4yS* and *y4yQ* genes, all in the same orientation. In the case of the *B. japonicum* USDA110 however there are two additional open reading frames (ORFs) between the *rhcV* and the *y4yQ* gene in the same orientation (Figure 4, Subgroup I). The third segment harbors the *rhcC2* gene usually between the *y4xI* and the *y4xK* genes.

Subgroup III of the *Rhc*-T3SS family includes the T3SS of *R. etli* strains CIAT652 (plasmid b) and CNF42 (plasmid d) [37]. The gene organization is very different from that of subgroup I in that there is no *rhcC2* gene, while the *rhcV* gene is in close proximity to the biggest segment. In the biggest segment the genes *y4yJ* (*hrpO/yscO/fliJ* homolog) and *nolB* are missing. Additional genes present in the subgroup III are coding for a HrpK-like protein (hypothetical translocator of the *Hrc-Hrp1* T3SS) and a HrpW-like protein.

Gene clusters of subgroup II of the *Rhc* T3SS family, represented by the the T3SS-2 of *Rhizobium* NGR234 (pNGR234b plasmid) [38] and the recently identified T3SS-2 gene clusters of the *P. syringae*, possesses various characteristics that classify them as intermediates between the T3SS subgroups I and III. On one hand, subgroup II clusters share the *sctO*, *sctD* and *sctC2* genes with subgroup I clusters and but not with subgroup III; on the other hand, some subgroup II clusters posses putative translocator genes present in subgroup III, but absent from subgroup I.

The T3SS-2 clusters of the *P. syringae* strains are essentially syntenic, with the exceptions of an IS element (insertion sequence element) being present between the Hrc_*II*_N and Hrp_*II*_O coding frames in the *P. syringae* pv phaseolicola 1448a cluster and the absence of a TPR (tetratricopeptide repeats) protein coding frame in the *P. syringae* pv oryzae str.1_6 cluster. The *Rhizobium* sp. NGR234 pNGR234b-plasmid borne cluster has two extended regions of synteny with those of the *P. syringae* strains. One is the region from *hrc*_*II*_*C*_*1*_ to *hrc*_*II*_*T*, [not including the IS element in the *P. syringae* pv phaseolicola 1448a cluster (see above)]. The other is the region from *hrp*_*II*_*Q* to PSPPH_2522 which, however, is inverted in the *Rhizobium* sp. NGR234 pNGR234b T3SS cluster relative to those in the pseudomonads. The coding frame for the RhcU/HrcU/YscU/FhlB homolog in the NGR234 cluster is transposed in relation to the *Pseudomonas* cluster (position which is maintained in the *R.etli* and *B. japonicum* clusters). In subgroup II of *Rhc-*T3SS gene clusters an *hrc*_*II*_*C2* gene can be identified in synteny to the subgroup I cluster. A common property of subgroups II and III of *Rhc*-T3SS gene clusters is the presence of *hrpK*-like genes.

Common to all *Rhc*-T3SS subgroups is the absence of a *hrpP/yscP* –like gene which usually resides between the *hrpO/yscO*-like gene and the *hrcQ/yscQ* homolog gene. A *hrpO/yscO-*like gene is absent from the subgroup III cluster. Subgroup I and III clusters maintain synteny with the *P. syringae* T3SS-2 clusters for most of the core T3SS ORFs. Finally, a gene coding for a HrpW homolog is found only in the *R. etli* clusters.

### Non-conserved T3SS proteins

#### The translocator of the P. syringae T3SS-2

A common feature of the *R. etli Rhc* T3SS (subgroup III) and the T3SS-2 of *P. syringae* pathovars (but not of the *Rhizobium* sp. NGR234 T3SS-2) is the presence of an ORF coding for a hypothetical translocator protein: The PSPPH_2540 locus of the *P. syringae* pv phaseolicola 1448a T3SS-2 codes for a large protein of 1106 residues. The C-terminal part of this protein (residues 421 – 1106) is homologous to the HrpK proteins of the *Hrc-Hrp1* T3SS family based on Psi-BLAST searches (25% identity with HrpK of *Erwinia amylovora*). HrpK shares low similarity with the putative translocator, HrpF, from *Xantomonas campestris* pv vesicatoria. Furthermore, the C-terminal part of the protein coded by PSPPH_2540 also possesses two predicted transmembrane α-helices comprising residues 879–898 and 1029–1047 (MEMSAT3 analysis). The subgroup I *Rhc* T3SS lacks a *hrpK* ortholog. The HrpK protein was initially identified as a component of the *Hrc-Hrp1* family of T3S systems [39]. Interestingly, the *R. etli* T3SS gene cluster possesses two copies of *hrpK*-like genes, plus an additional *hrpW*-like gene, coding for an Hrp-secreted protein homologous to class III pectate lyases which is absent from the *P. syringae* pv phaseolicola 1448a T3SS-2 gene cluster but present in the extremity of the *Hrc-Hrp1* gene cluster of *P. syringae* pv phaseolicola 1448a. These differences possibly suggest variations in the mode of interaction of these bacteria with their hosts.

#### The two unknown ORFs upstream of the rhcV gene in subgroup II Rhc-T3SS gene clusters

The choice of the *B. japonicum* USDA 110 T3SS as archetypal for subgroup I in the *Rhc* family (Figure 4) and for synteny comparisons with the subgroup II gene clusters, was based on the DNA segment encompassing *rhcV* (*y4yQ-y4yS*). The presence of two small open reading frames upstream of the *rhcV* gene and downstream of the *y4yQ* gene of the known *Rhizobium* T3SS resembled the case of the *P. syringae* pv phaseolicola 1448a T3SS-2 where loci PSPPH_2518 and PSPPH_2519 are found between the ORF coding for the SctV protein (RhcV/HrcV/LcrD/FlhA homolog) and the ORF coding for the SctD protein (HrpQ/YscD homolog).

The PSPPH_2519 locus, upstream of the *hrc*_*II*_*V* gene of *P. syringae* pv phaseolicola 1448a genome, encodes for a 112 long polypeptide with sequence similarities to the VscY protein of *Vibrio parahaemolyticus*, according to Psi-BLAST searches (E-value = 0.005). The *vscY* gene is located upstream of the *vcrD* gene and this synteny is also conserved in the *Ysc* T3SS gene cluster family. Proteins YscY, VscY and PSPPH_2519 all possess TPR repeats (Tetratricopeptide Repeats) as predicted by Psi-BLAST searches and fold recognition methods. YscY has been found to directly bind the YscX protein, a secreted component of the *Ysc* T3SS [40]. The *bll1801* locus of *B. japonicum* USDA110 encodes for a 142 long polypeptide with TPR repeats and sequence similarities to the AscY (*Aeromonas salmonicida*) and YscY proteins according to Psi-BLAST searches. The position of *bll1801* is likewise upstream of the *rhcV* gene in *B. japonicum* USDA110 T3SS gene cluster. A protein with the above characteristics could not be identified for the *R. etli* T3SS (subgroup III), however it is present in the T3SS-2 of *Rhizobium* NGR234.

#### Transcription regulators in P. syringae T3SS-2

The *Hrc-Hrp2* and the *Rhc* T3S (subgroup I) systems possess transcription regulators that belong to the AraC/XylS in contrast to the *Hrc-Hrp1* T3SS that depends on the alternative sigma factor HrpL. The known transcription factors are related to the T3SS regulation of AraC and LuxR/UhaP families of transcription regulators and characterized by two α-helix-turn-α-helix (HTH) motifs in a tetrahelical bundle.

**Figure 5:**
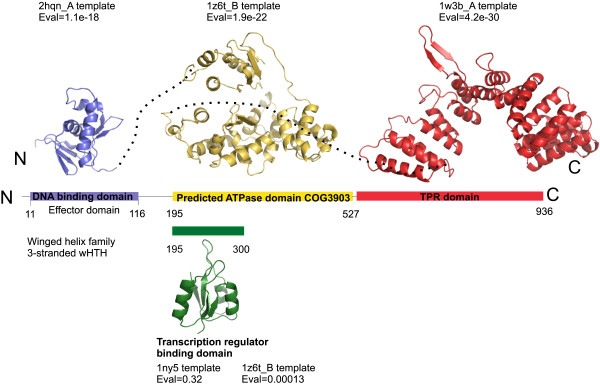
**Predicted PSPPH_2539 protein domain structure based on fold recognition analysis.** See text for details on the various structural templates used. Black dots connect the C-terminus of one threading domain with the N-terminus of the following domain. Residues 195–300 (green segment) are represented separately as an alternative fold for the N-terminal subdomain of the full length AAA^+^ ATPase domain (yellow).

However, the PSPPH_2539 locus of *P. syringae* T3SS-2 codes for a hypothetical transcription regulator with different characteristics. The N-terminal part of the hypothetical protein (Figure 5, blue-purple area) is predicted to adopt a structure similar to the DNA-binding domains of the PhoB transcription factor. The characteristic HTH motif is a common feature of transcription factors. Although the PSPPH_2539 ORF is annotated in the NCBI as a LuxR-type of transcription regulator, the choice of the DNA-binding domain of PhoB as a structural template indicates that PSPPH_2539 probably has an α-/β- doubly wound fold (distinguished by the presence of a C-terminal β-strand hairpin unit that packs against the shallow cleft of the partially open tri-helical HTH core) motif. Transcription factors are usually multidomain proteins, thus the assignment of PSPPH_2539 as a LuxR-type transcription regulator in the NCBI is probably due to full-length inadequate Psi-BLAST searches biased by the presence of Tetratricopeptide Repeats (TPR) in the large carboxyterminal domain.

The middle part of the protein (Figure 5, yellow area) was found homologous to the AAA^+^ ATPases (COG3903) based on fold-recognition algorithms and Psi-BLAST searches. These ATPases are associated with diverse cellular activities and are able to induce conformational changes in their targets [41]. In the context of the transcription process, AAA^+^ ATPase domains are involved in the remodeling of σ^54^ RNA polymerases. Especially the residues 195 to 300 probably possess the receiver or ligand binding domain of the hypothetical transcription factor (green area, Figure 5).

#### TPR-repeats proteins present in P. syringae T3SS-2

Apart from the PSPPH_2539 C-terminal domain, there are two more ORFs, PSPPH_2519 and PSPPH_2523, from the *P. syringae* pv phaseolicola 1448a T3SS-2 that are predicted to code for proteins that possess TPR domains. TPR domains are typically found in class II chaperones of T3S systems - chaperones of the translocators - as well as in transcriptional regulators of the T3S systems, e.g. the HrpB protein of *Ralstonia solanacearum*, HilA of *Salmonella enterica*[42] and SicA, of *Salmonella typhimurium* involved in the activations of T3SS virulence genes [43]. Proteins with TPR repeats also exist in the *Hrc-Hrp2* T3S system of *X. campestris* (HrpB2 protein) and in the T3S system of *Rhizobia* (e.g. the 182 residue long Y4yS protein). On the other hand, the *Hrc-Hrp1* system of *P. syringae* does not possess proteins with TPR repeats.

### DNA characteristics of the *P. syringae* T3SS-2 gene cluster

The T3SS-2 cluster of *P. syringae* pv phaseolicola 1448a is separated by 1.42 Mb from the well-characterized *Hrc-Hrp1* T3SS cluster in the main chromosome. Both clusters are located on DNA segments with GC content similar to their neighbouring areas. No sequences associated with HrpL-responsive promoters (characteristic for the regulation of the *Hrc-Hrp1* operons in *P. syringae* pathovars) were found in the T3SS-2 gene cluster [44] indicating a different way of regulation from the *Hrc-Hrp1* system. The ORF PSPPH_2539 that resides between the core genes and the *hrpK* homolog PSPPH_2540, codes for a hypothetical transcription regulator (Figure 4, 5). No *t*_*RNA*_ genes, however, have been found in the vicinity of this cluster, while two insertion sequence (IS) elements occur in the border and in the middle region of the T3SS-2 gene cluster (Figure 4).

The GC content of the T3SS-2 cluster in the *P. syringae* strains is close to the chromosome average (58–61%), which might suggest that it has been resident in the *P. syringae*’s genome for a long time [45]. The codon usage indexes (Additional file 7: Table S2) of the T3SS-2 cluster show the same degree of codon usage bias as the *hrc-hrp1* T3SS cluster of *P. syringae* pv phaseolicola 1448a. Furthermore, the GC content in the third coding position (GC3) of various genes across the T3SS-2 is close to the respective mean of the genome GC3, as in the case of *Hrc-Hrp1* (Additional file 7: Table S2). These equal GC levels could indicate an ancient acquisition of the T3SS-2 gene cluster by *P. syringae* that was lost in some of its strains. However the scenario of a more recent acquisition from a hypothetical donor with equal GC levels can not be excluded.

### Evidence for expression of the *P. syringae* T3SS-2

There are no reports so far for the expression or function of T3SS-2 in members of *P. syringae.* To obtain preliminary expression evidence of functional putative RNA transcripts, the *hrc*_*II*_*N* (*sctN*) and *hrc*_*II*_*C1* (*sctC*) from *P. syringae* pv phaseolicola 1448a were detected by RT-PCR in total RNA extracts from cultures grown in rich (LB) and minimal (M9) media, after exhaustive treatment with RNase-free DNase I (Supplier *Roche* Applied Science). Putative transcripts were detected under both growth conditions that were tested, using equal amounts of the extracted total RNA as an RT-PCR template. Interestingly, the detected transcript levels were remarkably higher in LB medium (Figure 3), compared to minimal (M9) medium, probably indicating that the genes are expressed in both cultivation conditions.

## Conclusions

Rhizobia are α-proteobacteria that are able to induce the formation of nodules on leguminous plant roots, where nitrogen fixation takes place with T3SS being one important determinant of this symbiosis [36,46,47]. Sequences of the symbiotic plasmids of *Rhizobium* strains NGR234 and *R. etli* CFN42 together with the chromosomal symbiotic regions of *B. japonicum* USDA110 and *Mesorhizobium loti* R7A have been recently reported [36-38]. An unusual feature of the *Rhizobium* strains NGR234 [38], is the presence of an additional T3SS gene cluster.

Members of the *P. syringae* species are gram negative plant-associated γ-proteobacteria that can exist both as harmless epiphytes and as pathogens of major agricultural crops [48-52]. Pathogenic varieties of this species utilize a *Hrc-Hrp1* T3SS to inject effector proteins and thus subvert signalling pathways of their plant hosts. This secretion system (*Hrc-Hrp1* T3SS) and its effector proteins are responsible for the development of the characteristic disease symptoms on susceptible plants and the triggering of the Hypersensitive Response (HR) in resistant plants [26,49,50,52].

Comparative genomics of closely related isolates or species of pathogenic bacteria provides a powerful tool for rapid identification of genes involved in host specificity and virulence [53]. In this work, we reported sequence similarity searches, phylogeny analysis and prediction of the physicochemical characteristics of the hypothetical T3SS-2 proteins, as well as gene synteny analysis of the T3SS-2 gene cluster in *P. syringae* pv phaseolicola 1448a, *P. syringae* pv oryzae str. 1_6 and *P. syringae* pv tabaci ATCC11528 in order to characterize this recently identified gene cluster. This analysis revealed that the T3SS-2 most closely resembles the T3SS of the *Rhc*-T3SS family. It further typifies a second discrete subfamily (subgroup II) within the *Rhc*-T3SS family in addition to the ones represented by the *R. etli* T3SS (subgroup III) and the known *Rhizobium*-T3SS (subgroup I). Usually, the presence of two T3SS gene clusters in the same genome is not the result of gene duplication inside the species but rather the result of independent horizontal gene transfers. This may reflect progressive coevolution of the plant patho/symbio-system to either colonize various hosts or interact with the plant in different disease/symbiotic stages.

In our phylogenetic analysis proteins encoded in the T3SS-2 cluster of *P. syringae* strains are grouped together with the *Rhizobium* NGR234 T3SS-2. This finding suggests the possibility of an ancient acquisition from a common ancestor for *Rhizobium* NGR234 T3SS-2 and the *P*. *syringae* T3SS-2. T3SSs of the *Rhizobium* family possesses a GC-content in same range (59-62%), a value lower than the chromosome average. Since the GC content of T3SS-2 is almost the same as that of the genome of the *P. syringae* strains, it is difficult to characterize the second T3SS gene cluster as a genomic island based solely on this criterion. However, the genome sequencing of two other members of *P. syringae* [pathovars *tomato* DC3000, *syringae* B728A] revealed the total absence of a T3SS-2 like cluster.

The T3SS-2 gene cluster found in *P. syringae* pv phaseolicola 1448a, *P. syringae* pv oryzae str.1_6, *P. syringae* pv tabaci and of *Rhizobium* sp. NGR234, is also present in *P. syringae* pv aesculi (strains NCPPB 3681 and 2250)[54], *P. syringae* pv savastanoi (str. NCPPB 3335) [55], *P. syringae* pv glycinea (strains: B076 and race 4) [56], *P. syringae* pv lachrymans str. M301315 (GenBank: AEAF01000091.1), *P. syringae* pv actinidiae str. M302091 (GenBank: AEAL01000073.1), *P. syringae* pv. morsprunorum str. M302280PT (GenBank: AEAE01000259.1) and *P. syringae* Cit 7 (GenBank: AEAJ01000620.1). This T3SS-2 defines a distinct lineage in the Rhc T3SS family of at least the same evolutionary age as the split between the NGR234 T3SS-2 from the other rhizobial T3SSs.

In light of these findings, there are two plausible scenarios. One is that *P. syringae* acquired the T3SS-2 cluster from an ancient donor which is common both to *P. syringae* and the *Rhizobium* sp. NGR234 T3SS-2, before the diversification of the *P. syringae* pathovars from each other, followed by subsequent loss from certain members of the group. Another scenario is that multiple horizontal transfers from hypothetical donors into selected pathovars/strains occurred after their diversification. The present data set does not allow us to consider whether the hypothesis of an earlier acquisition followed by subsequent loss from members such as *P. syringae* pv tomato DC3000 might be considered more likely than several independent acquisitions.

The genes *hrc*_*II*_*N* and *hrc*_*II*_*V* in *P. syringae* pv tabaci and *P. syringae* pv oryzae T3SS-2 clusters were split into at least two open reading frames in various positions suggesting possibly that they might be degenerate pseudogenes, while the *hrc*_*II*_*C2* gene in *P. syringae* pv tabaci is further split in two ORFs as well (Figure 4). However, this is not the case for the *P. syringae* pv phaseolicola 1448a, *P. syringae* pv savastanoi and *P. syringae* pv aesculi T3SS-2 where all these genes remain intact while *hrc*_*II*_*C1* and *hrc*_*II*_*N* transcripts were observed in *P. syringae* pv phaseolicola 1448a T3SS-2 case (Figure 4). Remarkably, the T3SS-2 genes expression was even higher in rich compared to minimal medium (Figure 3). Minimal media of slightly acidic pH are thought to simulate *in planta* conditions and promote expression of the *P. syringae* T3SS-1 and effectors [24,57,58]. Such genes typically possess conserved motifs (*hrp* boxes) in their promoter regions and are transcriptionally controlled by the alternative sigma factor HrpL. However, the T3SS-2 operons in the *P. syringae* pv phaseolicola 1448a genome do not appear to have *hrp* boxes like those found in T3SS-1 genes of *P. syringae* strains [27]. This suggests that *Psph* 1448a does restrict T3SS-2 expression to *in planta* conditions and the potential contribution of the T3SS-2 in *P. syringae* life cycle may not be connected with the phytopathogenic potential of this species. Further functional studies are thus needed to reveal the exact biological roles of this secretion system in bacterium-plant interactions or other aspects of the bacterial life cycle. Suppression of other secretion systems under the T3SS-1 inducing conditions has also been reported for the T6SS of *P. syringae* pv syringae B728a [59] as well as for the *P. aeruginosa* T3SS [60], which do not appear to play a role in plant pathogenesis [59,61,62].

Gene transfer between phylogenetically remote bacteria would be favored by colonization of the same environmental niche [63]. In nature, *Rhizobium* is normally viewed as a microbe that survives saprophytically in soil, in nitrogen fixing nodules of legumes or as endophytes in gramineous plants, for example field grown [64] and wild rice [65]. *P. syringae* pv phaseolicola 1448A and *P. syringae* pv oryzae str.1_6 are pathogens of the common bean and rice, respectively, while *Rhizobium* sp. NGR234 forms nitrogen fixing nodules with more legumes than any other microsymbiont [38]. Thus, there is ample opportunity for niche overlap between at least one of the *P. syringae* pathovars possessing T3SS-2 and *Rhizobium* sp. NGR234. At this point, a role for T3SS-2 in host-bacterium interactions for the rhizobia or the *P. syringae* strains possessing the system remains to be established and it is not obvious why these bacteria maintain a second T3SS gene cluster in their genome. Functional analysis and genome sequencing of more rhizobia that share common niches with *P. syringae* as well as the sequencing of more *P. syringae* pathovar genomes may shed light into these questions.

## Authors’ contributions

ADG performed research, helped draft the manuscript, analysed results and prepared figures. PFS, VEF, SNC and NM performed research, analysed results and critically appraised the manuscript. NJP and MK designed research, supervised work, organized financial support and critically appraised the manuscript. All authors read and approved the final manuscript.

## Supplementary Material

Additional file 1: Figure S1 Unrooted neighbor-joining phylogenetic tree of SctQ proteins of flagellar and non-flagellar T3S proteins. The tree was calculated by CLUSTALW (1.82) using bootstrapping (500 replicates) as a method for deriving confidence values for the groupings and was drawn by MEGA 4.0. Bootstrap values are indicated in each branching point. Scale bar represents numbers of substitution per site. The arrow indicates a possible position of root so that the tree will be compatible with the monophyly of the flagellar T3SS. Consistently with phylograms based on other conserved proteins of the *Pph* T3SS-2, the Hrc_*II*_Q polypeptide does not fall into any of the two *Hrc1/Hrc2* T3SS families but it is grouped with the *Rhc* family. Click here for file

Additional file 2: Figure S2. Unrooted neighboring joining tree including all known SctV T3SS families and the flagelar proteins. Bootstrap values are percentages of 500 repetitions taking place. Multiple alignment performed with ClustalW. Click here for file

Additional file 3: Figure S3 Evolutionary relationships of 250 HrcN/YscN/FliI proteins. A. The phylogram of 253 SctN sequences subdivided in seven main families, depicted with different colors and named according to [8], while the flagellum proteins are depicted in black. The evolutionary history was inferred as in case of Figure 2. B. The *Rhc* T3SS clade as derived from the phylogram in A, groups clearly the *P. syringae* Hrc_*II*_V sequences close to the Rhc_*II*_V protein of the *Rhizobium* sp. NGR234 T3SS-2. The values at the nodes are the bootstrap percentages out of 1000 replicates. The locus numbers or the protein accession number of each sequence is indicated. Click here for file

Additional file 4: Table S1 Sequence comparisons of T3SS-2 proteins with proteins from from subgroups I-III of *Rhc* T3SS gene clusters. Percentage identities of various T3SS proteins in comparison to the *Pph* T3SS-2 proteins. *Pph* T3SS-2 cluster shares a higher degree of common genes with T3SS-2 of *Rhizobium* sp. NGR234 than with *Rhc* T3SS gene clusters of subgroup I or III. Shading in grayscale is according to percentage identity. Click here for file

Additional file 5: Figure S4 Multiple alignements with ClustalW version 1.8 [19] for A) RhcC1 proteins (ref|YP 274720.1| Hrc*II*C1 *Pseudomonas syringae* pv. phaseolicola 1448a], ref|ZP 04589253.1| Hrc*II*C1 *Pseudomonas syringae* pv. oryzae str. 1_6], ref|YP 002824487.1| Rhc*II*C *Rhizobium* sp. NGR234], ref|NP 444156.1| NolW *Rhizobium* sp. NGR234], ref|NP 106861.1| NOLW *Mesorhizobium loti* MAFF303099], ref|NP 768451.1| RhcC1 *Bradyrhizobium japonicum* USDA 110] and B) RhcC2 proteins (ref|ZP 04589255.1|Hrp*II*C2 *Pseudomonas syringae* pv. oryzae str. 1_6], ref|YP 002824481.1| Rhc*II*C2 *Rhizobium* sp. NGR234], ref|NP 106858.1| RhcC2 *Mesorhizobium loti* MAFF303099], ref|NP 768482.1| RhcC2 *Bradyrhizobium japonicum* USDA 110] and ref|NP 444146.1| Y4xJ *Rhizobium* sp. NGR234]. Visualization of the alignment was performed in http://www.bioinformatics.org/sms2/color_align_cons.html. Click here for file

Additional file 6: Figure S5 Sequence analysis for HrpO-like proteins. The analysis of PSPPH_2532 (Hrp*II*O) indicates that this hypothetical protein belongs to the HrpO/YscO/FliJ family of T3SS proteins [5,33]. The same is evident for the sequence annotated as RhcZ in the T3SS-2 of *Rhizobium* sp. NGR342. Residues predicted in α-helical conformation are indicated in yellow and unfolded regions in red. Green areas indicate ordered regions. Residues for which a high propensity for coiled-coil formation is predicted are indicated in blue rectangular. Here α-helix prediction was performed with PsiPRED, disordered prediction with FOLDINDEX and coiled coils prediction with COILS. Accession numbers or loci numbers are: AAC25065 (HrpO), P25613 (FliJ), AAB72198 (YscO), PSPPH_2532 (Hrp*II*O), NGR_b22960 (RhcZ), NGR234_462 (Y4yJ). Click here for file

Additional file 7: Table S2 Codon Usage Bias Table. Click here for file
