# Supplementary material for: Phylogenetic analysis of a gene cluster encoding an additional, rhizobial-like type III secretion system that is narrowly distributed among Pseudomonas syringae strains
Source: BMC Microbiol. 2012 Sep 2;12:188. doi: 10.1186/1471-2180-12-188 (PMC3574062; doi:10.1186/1471-2180-12-188)
Supplement: Additional file 1: Figure S1 — Unrooted neighbor-joining phylogenetic tree of SctQ proteins of flagellar and non-flagellar T3S proteins. The tree was calculated by CLUSTALW (1.82) using bootstrapping (500 replicates) as a method for deriving confidence values for the groupings and was drawn by MEGA 4.0. Bootstrap values are indicated in each branching point. Scale bar represents numbers of substitution per site. The arrow indicates a possible position of root so that the tree will be compatible with the monophyly of the flagellar T3SS. Consistently with phylograms based on other conserved proteins of the Pph T3SS-2, the HrcIIQ polypeptide does not fall into any of the two Hrc1/Hrc2 T3SS families but it is grouped with the Rhc family. [file 1471-2180-12-188-S1.pdf]

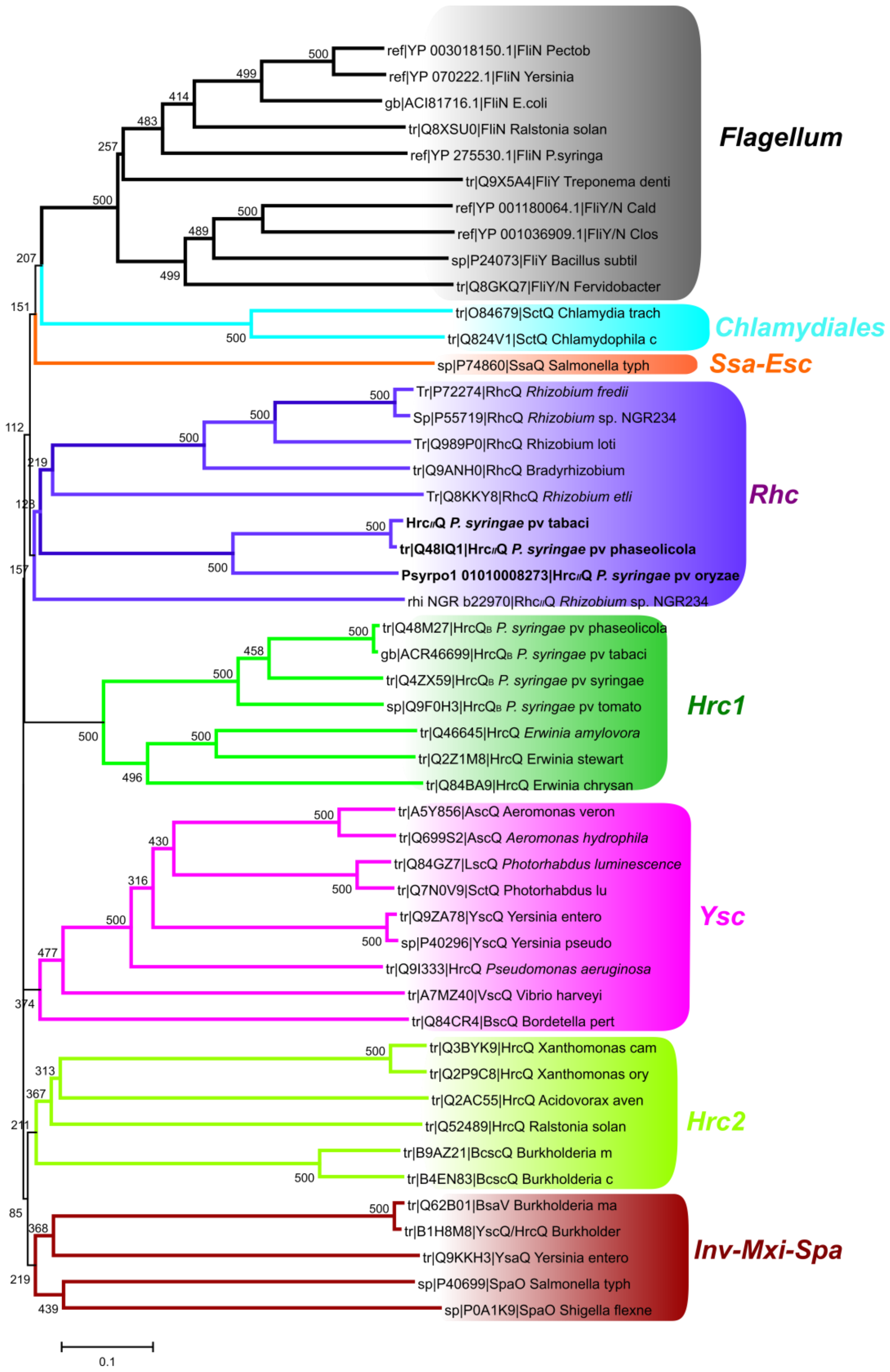

Additional file 1. Figure S1 :

Unrooted neighbor-joining phylogenetic tree of SctQ proteins of flagellar and non-flagellar T3S proteins. The tree was calculated by CLUSTALW (1.82) using

bootstrapping (500 replicates) as a method for deriving confidence values for the groupings and was drawn by MEGA 4.0. Bootstrap values are indicated in each branching point. Scale bar represents numbers of substitution per site. The arrow indicates a possible position of root so that the tree will be compatible with the monophyly of the flagellar T3SS. Consistently with phylograms based on other conserved proteins of the *Pph* T3SS-2, the $Hrc_{II}Q$ polypeptide does not fall into any of the two *Hrc1/Hrc2* T3SS families but it is grouped with the *Rhc* family.
