# Supplementary material for: Phylogenetic analysis of a gene cluster encoding an additional, rhizobial-like type III secretion system that is narrowly distributed among Pseudomonas syringae strains
Source: BMC Microbiol. 2012 Sep 2;12:188. doi: 10.1186/1471-2180-12-188 (PMC3574062; doi:10.1186/1471-2180-12-188)
Supplement: Additional file 2: Figure S2. — Unrooted neighboring joining tree including all known SctV T3SS families and the flagelar proteins. Bootstrap values are percentages of 500 repetitions taking place. Multiple alignment performed with ClustalW. [file 1471-2180-12-188-S2.pdf]

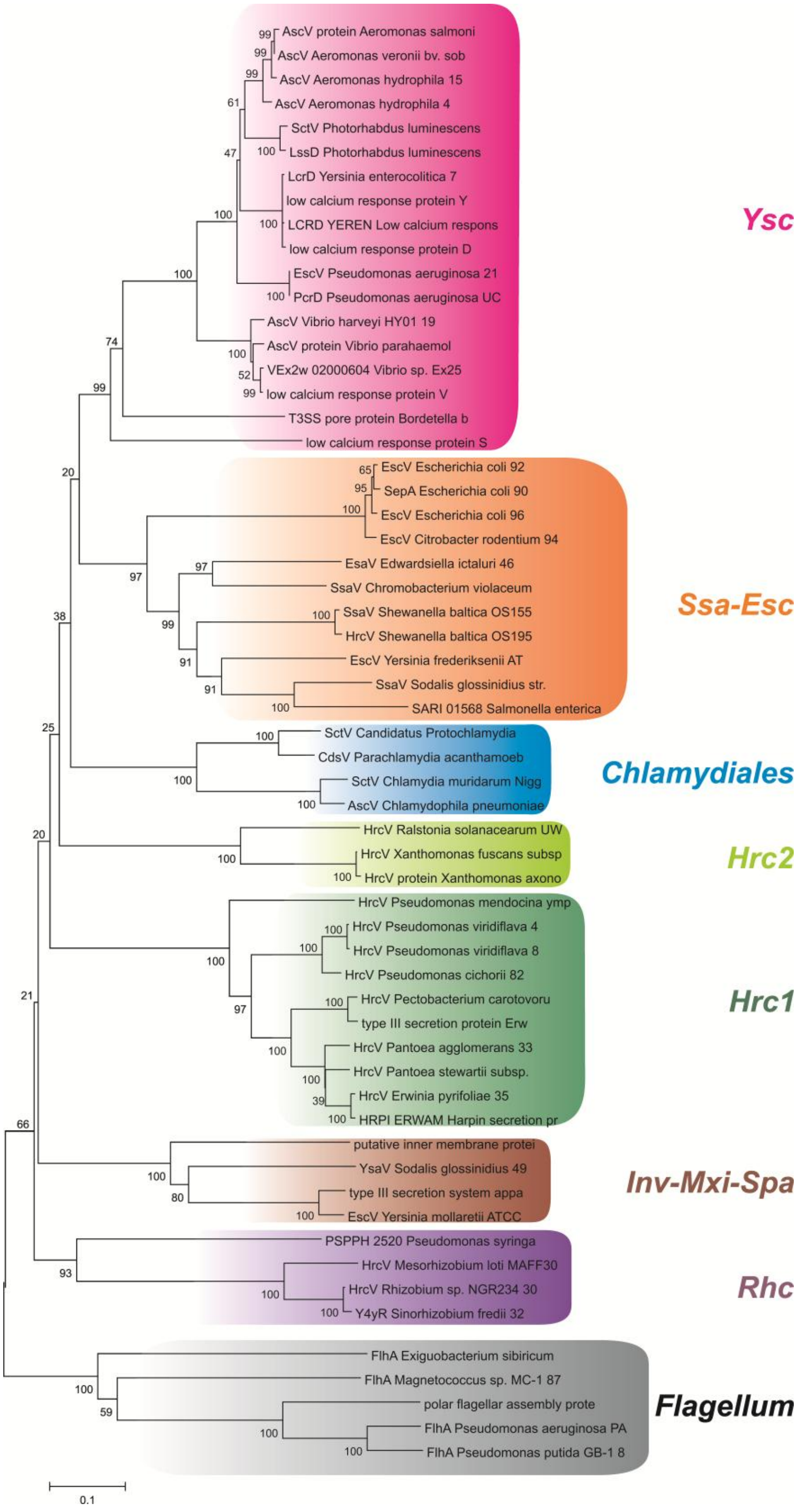

Additional file 2 . Figure S2 :

Unrooted neighboring joining tree including all known SctV T3SS families and the flagelar proteins. Bootstrap values are percentages of 500 repetitions taking place. Multiple alignment performed with ClustalW.
