# Supplementary material for: Phylogenetic analysis of a gene cluster encoding an additional, rhizobial-like type III secretion system that is narrowly distributed among Pseudomonas syringae strains
Source: BMC Microbiol. 2012 Sep 2;12:188. doi: 10.1186/1471-2180-12-188 (PMC3574062; doi:10.1186/1471-2180-12-188)
Supplement: Additional file 3: Figure S3 — Evolutionary relationships of 250 HrcN/YscN/FliI proteins. A. The phylogram of 253 SctN sequences subdivided in seven main families, depicted with different colors and named according to [8], while the flagellum proteins are depicted in black. The evolutionary history was inferred as in case of Figure 2. B. The Rhc T3SS clade as derived from the phylogram in A, groups clearly the P. syringae HrcIIV sequences close to the RhcIIV protein of the Rhizobium sp. NGR234 T3SS-2. The values at the nodes are the bootstrap percentages out of 1000 replicates. The locus numbers or the protein accession number of each sequence is indicated. [file 1471-2180-12-188-S3.pdf]

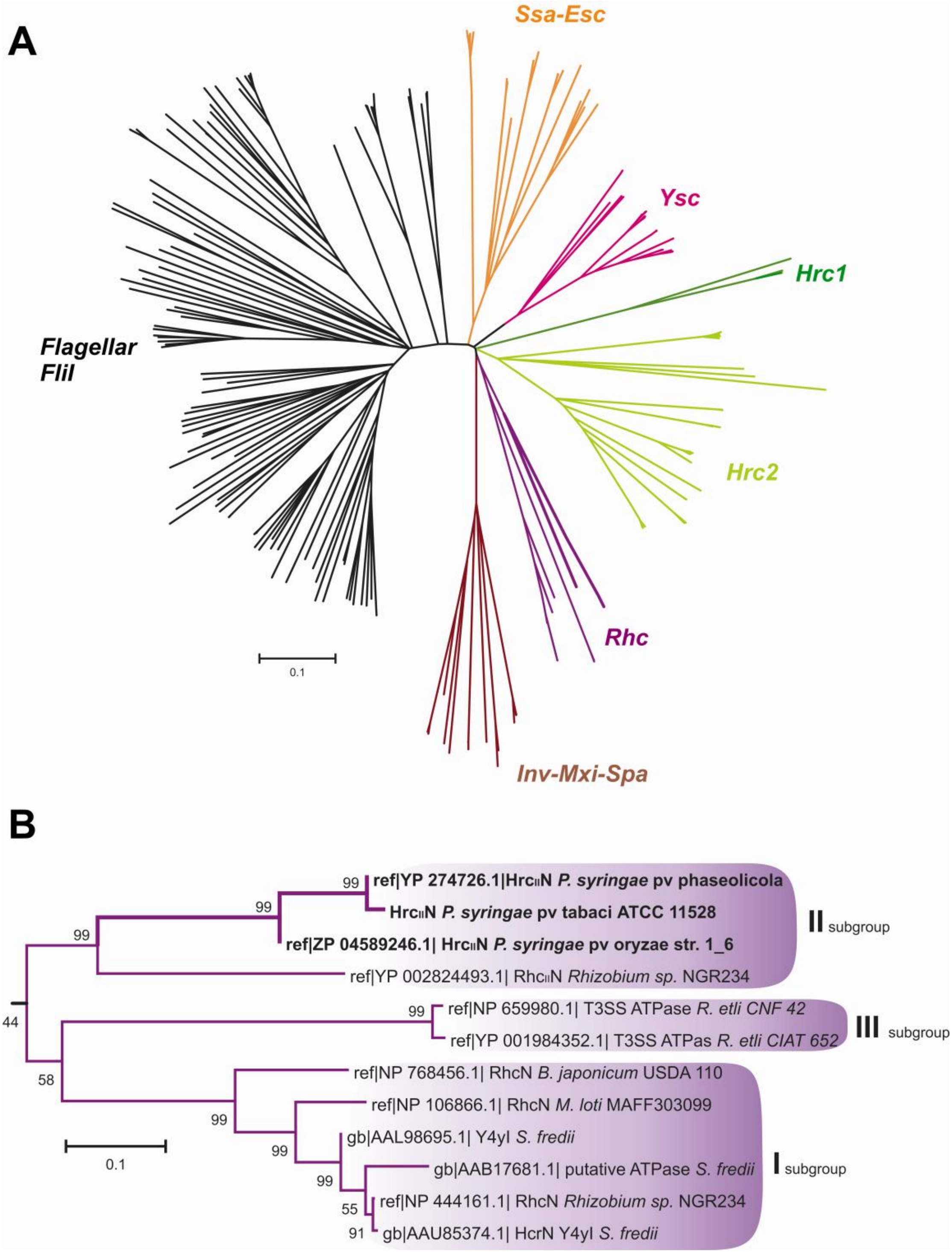

Additional file 3. Figure S3 : Evolutionary relationships of 250 HrcN/YscN/FliI proteins

A. The phylogram of 253 SctN sequences subdivided in seven main families, depicted with different colors and named according to [8], while the flagellum proteins are depicted in black. The evolutionary history was inferred as in case of Fig. 2

B. The *Rhc* T3SS clade as derived from the phylogram in A, groups clearly the *P. syringae* Hrc$_{II}$V sequences close to the Rhc$_{II}$V protein of the *Rhizobium* sp. NGR234 T3SS-2. The values at the nodes are the bootstrap percentages out of 1000 replicates. The locus numbers or the protein accession number of each sequence is indicated.
