# Supplementary material for: Phylogenetic analysis of a gene cluster encoding an additional, rhizobial-like type III secretion system that is narrowly distributed among Pseudomonas syringae strains
Source: BMC Microbiol. 2012 Sep 2;12:188. doi: 10.1186/1471-2180-12-188 (PMC3574062; doi:10.1186/1471-2180-12-188)
Supplement: Additional file 4: Table S1 — Sequence comparisons of T3SS-2 proteins with proteins from from subgroups I-III of Rhc T3SS gene clusters. Percentage identities of various T3SS proteins in comparison to the Pph T3SS-2 proteins. Pph T3SS-2 cluster shares a higher degree of common genes with T3SS-2 of Rhizobium sp. NGR234 than with Rhc T3SS gene clusters of subgroup I or III. Shading in grayscale is according to percentage identity. [file 1471-2180-12-188-S4.pdf]

A

```
NolW RhizobiumT3SS-1 ----MPTTPIPFTPLHMFRRLLCVGLFLFAGIHTTLGATLPLPSTSYKYTVLDQDLSAALQEFGNNLKISVNISAEVKGR 76
NolW M. loti         ------------------MRLLCAGFFLSAGTNGTLGVPLSLSKTPYRYTVLDQDISEALQQFGNNLNIRVNISAEVKGR 62
RhcC1 B. japonicum   MSGRRQGMLARSTVVHILKRIFCAGVLICVGILRTSAASLSLPPAPYSYTVLDQDLAAALQEFGNNLNIRVNVSADVRGR 80
HrcIIC1 Pph T3SS-2   ------MKHFRADPPVSKLLMLILLCLLSGVLKAASERQPDWFSEPYAYVLVDQDIRGALTEFGQHLGLIVVFSEKVRGN 74
HrcIIC1 Psory T3SS-2 -------------------------------------------------------------------------------- 0
RhcIIC1 NGR234T3SS-2 ---MRTIGTFAMKMVMFTGRLCVFAIATLAATAASFAEAPRWYDSRYSYVLINQDVRDALKEFGRNLSLPVVLSDRIRGQ 77

NolW RhizobiumT3SS-1 IRGRIPELSPREFLDRLTDLYDLQWYYDGVVLYVSAAKEAQTRMLVLSSVHFSAFKLALDKLDISDERYPVRPAPGNGLV 156
NolW M. loti         IRGSMPDLPPREFLDRLANMYGLQWYYDGLVLYVSAAKESQTRMLVLTSIRFDTFKGALDKLEISDDRYVVRPAPGDGLV 142
RhcC1 B. japonicum   IRGRMPDLPPREFLDRLTALYNLQWYYDGLVLYISAAQEAQSRLIVLNPISFDALKAALDALNISDERYIVKPAPGEGLI 160
HrcIIC1 Pph T3SS-2   ARGTVRGEDAGEFLTRLCDANQLSWYFDGNVLHIAGADEVATRVFDLQGPRLEELQRYMARLEVSGQPMSSRVSPDSDSL 154
HrcIIC1 Psory T3SS-2 -RGTVRGKTAGEFLGHLCDANQLSWYFDGNVLHIAGADEVGTRVFDLQGAPLQELQDYLTRLEVSGQPMSSRVSPDNDSL 79
RhcIIC1 NGR234T3SS-2 VRGEIRAATAGDFLDRLTQANGLIWYFDGSILHINTSDEFSTQIIDIGRANGRSVIDEIERLDLMDGRFSVRATTNAPAL 157

NolW RhizobiumT3SS-1 LVSGPPRFMALIEQTLNGLLAVAQAQ-PRATDTPARESVMVLFRGSSTTVVRGGRPEVFYTSEMLPENDDGGKAELSKK 234
NolW M. loti         LVSGPPRFTALVEQTFNGLVAEAQGQ-PRVHETPTSESVLTLFRGSSTMVVRNGLPEAAYSSDVPQQDGVGGKPEPRQK 220
RhcC1 B. japonicum   LASGPPRFVALVDQTLKGLVAEAQARRSPAAERSQHESVLMLFRGSSSTVFRDGRPEA--SPETPLHGGAVREAGPGQK 237
HrcIIC1 Pph T3SS-2   FVSGPPAWLAQIQHHVDRQPAAEVAP-------VVRERGVRVFRGG-----VVTQVATDRQ------------------ 203
HrcIIC1 Psory T3SS-2 FVSGPPAYLAQIQQHVDRQPVAEAAP-------VVRERGVRVFRGG-----VVTQVATDRQ------------------ 128
RhcIIC1 NGR234T3SS-2 RVSGPPPFIAMVKQVAASVQPPPATQ-------VDDPR-VRIFRGGQRDEVTADLVETDRQAAPANRKKTKSPAGEQ-- 226
```

B

```
RhcC2 M. loti            -----------------------------MQDQGAPRAASNSIDGTLNLSSSLGKTVHLPAPATTIFVADPTIADYQAAS 51
Y4xJ RhizobiumT3SS-I    ----------------------------------MPRAAPNSINATLNLSSSLGKTVHLPAPAATIFVADPTIADYQAPS 46
RhcC2 B. japonicum      ----------------------------------MPRAAPGSTTGTLNITSSQGKTVHLSAPAATIFVADPAIADYQAPS 46
HrpIIC2 Pph 1448A       ---------MTSVSVIRMFLVGVGALLIGTGSFAENIAKGAKG--TVDLAIGEGRVLHFSAPVDSVMVAEPGIADLQVVS 69
HrpIIC2 Psory 1_6       --------------MIRVLFLALGALLLAGGANAQTIAKGAEG--TIDLAIGEGRVLHFSAPVDSVMVAEPGIADLQVVS 64
RhcIIC2 Rhizobium 2     MLVLRSLRPVRTGKVFLLILLTVLAVHCALPSASAQVKVDAINGQVLELTVGEGTILRFDEPVESVFLADTAIADVRVVS 80

RhcC2 M. loti            NTTIFVFGKKSGRTSLFALDDKGEALAALRIVVTQPIEELRAMLMDQVGDSSIQVSYTPRGAILSGTAPNAEVADTAKRV 131
Y4xJ RhizobiumT3SS-I    NRTIFVFGKKFGRTSLFALDENGEALAELHVVVTQPIADLRAMLRDQVGDYPIHVSYTPRGAILSGTAPNAEVVDIAKRV 126
RhcC2 B. japonicum      SSTIFVFGKKSGRTSLFALNENGEALAELRIVVTQPLEDLRAALKAEVGDYPIQVSYTPRGAILSGIAPNADVVEAARKV 126
HrpIIC2 Pph 1448A       PGVIYVFGKAAGQTSLIALDSDGRETAALSLAVSSGTAAVTRPLKALHPQSQARINASGSRVIASGSVQDVGEATDLNAL 149
HrpIIC2 Psory 1_6       PGVIYVFGKTAGQTSLIALDSEGRETAALSLAVSSGTAAVTRPLKALHPQSRARISASGNRVIASGSVQDVGEATDLNAL 144
RhcIIC2 Rhizobium 2     PGVVYIYATKTGDTNLIALSADQMTRGTVQVRVSGNPRAAEQSALALQPTTRVDISLFGGQYVGTGQTRNVGEAMDMESV 160

RhcC2 M. loti            TEQYLGDGAQVVNNIKVAGSLQVNLSVRVAEVSRSAMKALGVNLSAFGQIDNFRVGLLSGGGTGSGAAQGGG-TAGIGFN 210
Y4xJ RhizobiumT3SS-I    TEQFLGDGAPIVNNIKVAGSLQVNLSVRVAEVSRSGLKALGINLSAFGQFGNFKVGVLNRGAGLGSATGSGG-TAEIGFD 205
RhcC2 B. japonicum      TEQFVGAGAPVVNKIQVAGSLQVNLSVRVAEVSRTAVKDLNINFTASGPNG---AFLATGKPGGSGRAGGGG-TIGIGFS 202
HrpIIC2 Pph 1448A       LSTEGQNFQSTVNSATYAGSAQVNIRVRFAEVSRSELLRYGVNWNALFNNGTFSFGLLTGGALAADAAGGASNVISAGLA 229
HrpIIC2 Psory 1_6       LSSEGQNFQSSVNSATYAGAAQVNIRVRFAEVSRSELLRYGVNWNALFNNGTFSFGLLTGGSLAADAASGASNVISAGLT 224
RhcIIC2 Rhizobium 2     LQSYSTPDRPALNNTTIAGSNQVNIRVRFAEVARNELTRFGIDWSLFVNSGSFSFGIVR----TGGASNEDDTGIAIGAR 236

RhcC2 M. loti            NGAVNIGAVLDALAKEHIASVLAEPNLTAMSGETASFLAGGEFPIPVLQENKQVSVEFRHFGVSLEFVPTVLNNNRINIH 290
Y4xJ RhizobiumT3SS-I    NDAVSVGAVLDALAKEHIASVLAEPNLTAMSGETASFLAGGEFPIPVLQENGQTSVEFRRFGVSLEFVPTVLDNNLINIH 285
RhcC2 B. japonicum      TGNINLSAVLDALASEHLASILAEPNLTAMSGEAASFLAGGEFPIPVMQDNRQVSVQFRQFGVSLEFVPTVLSNNQIIVR 282
HrpIIC2 Pph 1448A       SGNVNIDAMLEALQSNGVLEVLAEPNITAMTGQTASFLAGGEVAVPVPVNREVVGIEYKPYGVSLLFSPTLLPNGRIALQ 309
HrpIIC2 Psory 1_6       SGNVNIDAMLEALQSNGVLEVLAEPNITAMTGQTASFLAGGEVAVPVPVNREVVGIEYKPYGVSLLFSPTLLPNGRIALQ 304
RhcIIC2 Rhizobium 2     GDHVNVNVLLDALQANGILTILAEPNITAVTGQTASFLAGGEIPVPVPAGDGQIGIEYKQFGVSLQFTPTLLPNNRIALQ 316

RhcC2 M. loti            VKPEVSELSSQGAVQINGISVPAVSTRRADTVVELASGQSFAIGGLIRRNVNNNVSAFPWLGEMPILGALFRSSSFQKEE 370
Y4xJ RhizobiumT3SS-I    VKPEVSELSLQGAVQVNGIAVPAVSTRRADTVVELASGQSFVIGGLIRRNVNNDISAFPWLGRIPILGALFRSSSFQKEE 365
RhcC2 B. japonicum      VKPEVSELSTEGEVKINGMAVPALSTRRASTVVELASGQSFAIGGLIRRNFNTDIGEFPWLGDVPILGALFRSSSFQKRE 362
HrpIIC2 Pph 1448A       VRPEVSSLMSTTTLDVNGYQVPSFRVRRADTRVEVGSGQTFAIAGLFQRESSQDMDKVPMLGDMPILGNLFRSKRFQRNE 389
HrpIIC2 Psory 1_6       VRPEVSSLMSTTTLDVNGYQVPSFRVRRADTRVEVGSGQTFAIAGLFQRESSQDMDKVPMLGDMPILGNLFRSKRFQRNE 384
RhcIIC2 Rhizobium 2     VRPEVSSVSQDSVVSISGLVVPSLRIRRADTAVEVGSGQTFAIAGLFQRQESQTLNKTPVVGDVPILGELFKSKRFQRNE 396

RhcC2 M. loti            SELIILVTPYIVKPGSSPNQMSAPTDRMAPALDD------PPADPPRGRAAARTGAPGAKRGRGFIIQ 432
Y4xJ RhizobiumT3SS-I    SELVILVTPYIVRPGSNPNQMSAPTDRMAPALG----------TPPRARAAISTDAPSVKGDLGFIIE 423
RhcC2 B. japonicum      TELVIVVTPYIVRPGSNPSQISIPTNRIAPPSDAGRILTNTVARPPQGRDAPRASAPGLTGNAGFIIE 430
HrpIIC2 Pph 1448A       TELVILITPYLVEP-VKDRVVATPLDKQRAASTAS-------------------AGPRSGGAFVFYMN 437
HrpIIC2 Psory 1_6       TELVILITPYLVEP-VKERVAATPLDKQRATSTAS-------------------AGPRSGGAFGFYMN 432
RhcIIC2 Rhizobium 2     TELVILITPYLVTP-TSERNMKTPLDSPAGAITSPR-----------------KKAKPAVNKGYGFYVE 447
```

Additional File 4. Figure S4 :

Multiple alignements with ClustalW version 1.8 [19] for A) RhcC1 proteins (ref|YP 274720.1| Hrc$_{II}$C1 [*Pseudomonas syringae* pv. phaseolicola 1448a], ref|ZP 04589253.1| Hrc$_{II}$C1 [*Pseudomonas syringae* pv. oryzae str. 1_6], ref|YP 002824487.1| Rhc$_{II}$C [*Rhizobium* sp. NGR234], ref|NP 444156.1| NolW [*Rhizobium* sp. NGR234], ref|NP 106861.1| NOLW [*Mesorhizobium loti* MAFF303099], ref|NP 768451.1| RhcC1 [*Bradyrhizobium japonicum* USDA 110] and B) RhcC2 proteins (ref|ZP 04589255.1| Hrp$_{II}$C2 [*Pseudomonas*

syringae pv. oryzae str. 1_6], ref|YP 002824481.1| Rhc$_{II}$C2 [*Rhizobium* sp. NGR234], ref|NP 106858.1| RhcC2 [*Mesorhizobium loti* MAFF303099], ref|NP 768482.1| RhcC2 [*Bradyrhizobium japonicum* USDA 110] and ref|NP 444146.1| Y4xJ [*Rhizobium* sp. NGR234]. Visualization of the alignment was performed in http://www.bioinformatics.org/sms2/color_align_cons.html.
