# Supplementary material for: Phylogenetic analysis of a gene cluster encoding an additional, rhizobial-like type III secretion system that is narrowly distributed among Pseudomonas syringae strains
Source: BMC Microbiol. 2012 Sep 2;12:188. doi: 10.1186/1471-2180-12-188 (PMC3574062; doi:10.1186/1471-2180-12-188)
Supplement: Additional file 5: Figure S4 — Multiple alignements with ClustalW version 1.8 [19] for A) RhcC1 proteins (ref|YP 274720.1| HrcIIC1 Pseudomonas syringae pv. phaseolicola 1448a], ref|ZP 04589253.1| HrcIIC1 Pseudomonas syringae pv. oryzae str. 1_6], ref|YP 002824487.1| RhcIIC Rhizobium sp. NGR234], ref|NP 444156.1| NolW Rhizobium sp. NGR234], ref|NP 106861.1| NOLW Mesorhizobium loti MAFF303099], ref|NP 768451.1| RhcC1 Bradyrhizobium japonicum USDA 110] and B) RhcC2 proteins (ref|ZP 04589255.1|HrpIIC2 Pseudomonas syringae pv. oryzae str. 1_6], ref|YP 002824481.1| RhcIIC2 Rhizobium sp. NGR234], ref|NP 106858.1| RhcC2 Mesorhizobium loti MAFF303099], ref|NP 768482.1| RhcC2 Bradyrhizobium japonicum USDA 110] and ref|NP 444146.1| Y4xJ Rhizobium sp. NGR234]. Visualization of the alignment was performed in http://www.bioinformatics.org/sms2/color_align_cons.html. [file 1471-2180-12-188-S5.pdf]

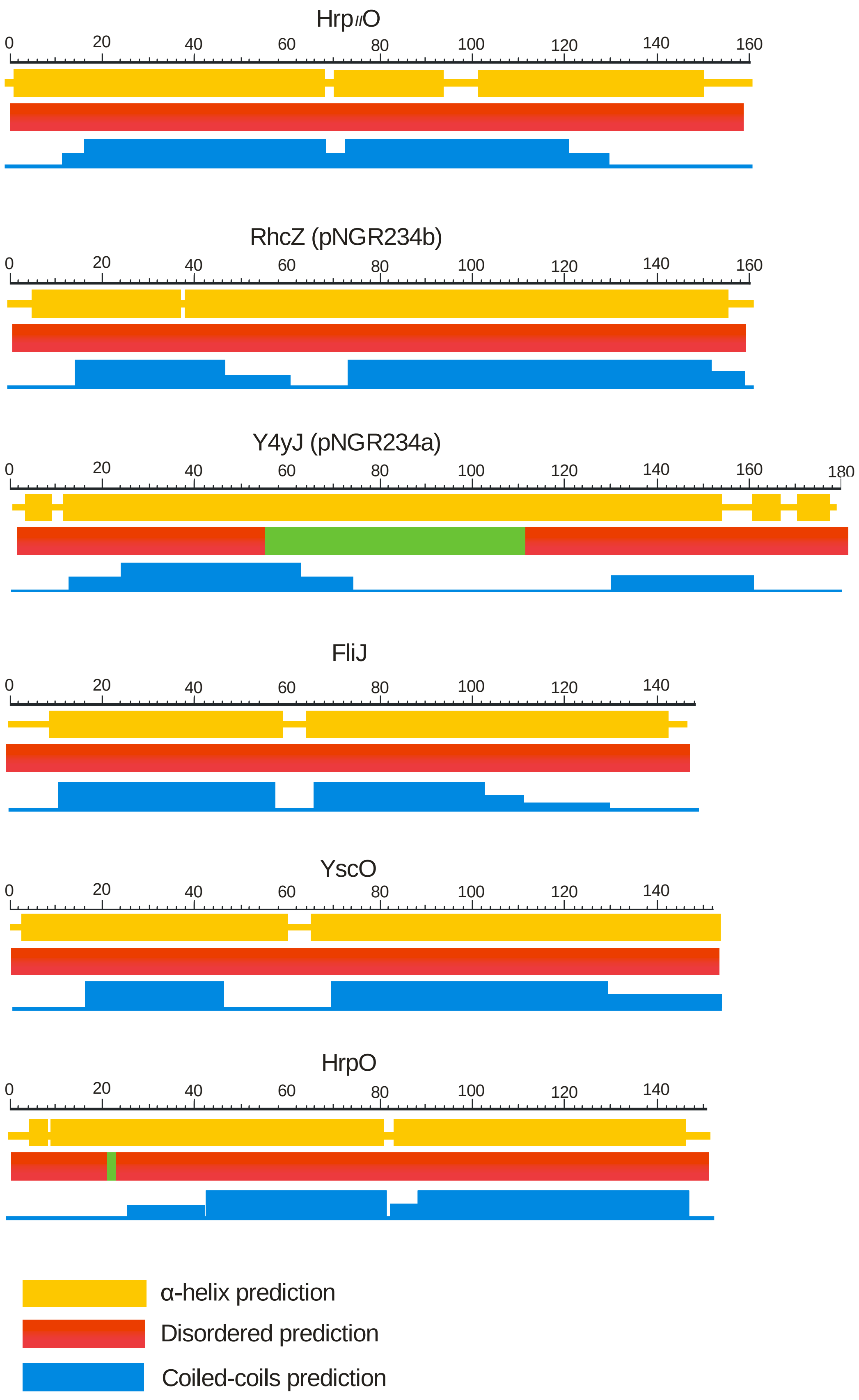

Additional File 5. Figure S5 :

Sequence analysis for HrpO-like proteins. The analysis of PSPPH_2532 ($Hrp_{II}O$) indicates that this hypothetical protein belongs to the HrpO/YscO/FliJ family of T3SS proteins [5, 32]. The same is evident for the sequence annotated as RhcZ in the T3SS-2 of *Rhizobium* sp. NGR342. Residues predicted in α-helical conformation are indicated in yellow and unfolded regions in red. Green areas indicate ordered regions. Residues for which a high propensity for coiled-coil formation is predicted are indicated in blue rectangular. Here α-helix prediction was performed with PsiPRED, disordered prediction with FOLDINDEX and coiled coils prediction with COILS. Accession numbers or loci numbers are: AAC25065 (HrpO), P25613 (FliJ), AAB72198 (YscO), PSPPH_2532 ($Hrp_{II}O$), NGR_b22960 (RhcZ), NGR234_462 (Y4yJ).
