# Supplementary material for: Phylogenetic analysis of a gene cluster encoding an additional, rhizobial-like type III secretion system that is narrowly distributed among Pseudomonas syringae strains
Source: BMC Microbiol. 2012 Sep 2;12:188. doi: 10.1186/1471-2180-12-188 (PMC3574062; doi:10.1186/1471-2180-12-188)
Supplement: Additional file 6: Figure S5 — Sequence analysis for HrpO-like proteins. The analysis of PSPPH_2532 (HrpIIO) indicates that this hypothetical protein belongs to the HrpO/YscO/FliJ family of T3SS proteins [5,33]. The same is evident for the sequence annotated as RhcZ in the T3SS-2 of Rhizobium sp. NGR342. Residues predicted in α-helical conformation are indicated in yellow and unfolded regions in red. Green areas indicate ordered regions. Residues for which a high propensity for coiled-coil formation is predicted are indicated in blue rectangular. Here α-helix prediction was performed with PsiPRED, disordered prediction with FOLDINDEX and coiled coils prediction with COILS. Accession numbers or loci numbers are: AAC25065 (HrpO), P25613 (FliJ), AAB72198 (YscO), PSPPH_2532 (HrpIIO), NGR_b22960 (RhcZ), NGR234_462 (Y4yJ). [file 1471-2180-12-188-S6.pdf]

	Pph 1448A T3SS-2	*Pstab* T3SS-2	*Psory* T3SS-2	*Rhizobium* NGR234 T3SS-2	*Rhizobium* NGR234 T3SS-1	*B. japonicum* T3SS	*R. etli* CNF42 T3SS
**Hrp*II*Q**	PSPPH_2517	95%	62%	32%	24%	25%	
	PSPPH_2518	100%	85%	36%			
	PSPPH_2519	97%	86%	48%			
**Hrc*II*V**	PSPPH_2520	98%	77%	46%	40%		
**Hrc*II*C2**	PSPPH_2521	99%	95%	51%	40%	38%	
	PSPPH_2522	97%	82%	37%			
**TPR protein**	PSPPH_2523	98%		38%			
**Hrc*II*C1**	PSPPH_2524	97%	87%	40%	32%	32%	
	PSPPH_2525	94%	86%				
	PSPPH_2526	100%	87%	39%			
**Hrc*II*J**	PSPPH_2527	97%	94%	58%	54%	54%	32%
**NolU homolog**	PSPPH_2528	96%	70%	32%	28%	30%	16%
**Hrp*II*E**	PSPPH_2529	99%	84%	36%	28%	31%	27%
**Hrc*II*N**	PSPPH_2530	96%	91%	62%	52%	53%	51%
**Hrp*II*O**	PSPPH_2532	98%	63%				
**Hrc*II*Q**	PSPPH_2534	98%	65%	26%	31%	32%	30%
**Hrc*II*R**	PSPPH_2535	99%	98%	71%	56%	50%	43%
**Hrc*II*S**	PSPPH_2536	98%	97%	76%	39%		
**Hrc*II*T**	PSPPH_2537	100%	98%	50%	33%	33%	30%
**Hrc*II*U**	PSPPH_2538	99%	91%	46%	36%	38%	30%
**transription regulator**	PSPPH_2539	95%	80%				
**Hrp*II*K**	PSPPH_2540	98%	85%				28%

Additional file 6. Table S1 :

Sequence comparisons of T3SS-2 proteins with proteins from from subgroups I-III of *Rhc* T3SS gene clusters. Percentage identities of various T3SS proteins in comparison to the *Pph* T3SS-2 proteins. *Pph* T3SS-2 cluster shares a higher degree of common genes with T3SS-2 of *Rhizobium* sp. NGR234 than with *Rhc* T3SS gene clusters of subgroup I or III. Shading in grayscale is according to percentage identity.
