# Supplementary material for: Phylogenetic analysis of a gene cluster encoding an additional, rhizobial-like type III secretion system that is narrowly distributed among Pseudomonas syringae strains
Source: BMC Microbiol. 2012 Sep 2;12:188. doi: 10.1186/1471-2180-12-188 (PMC3574062; doi:10.1186/1471-2180-12-188)
Supplement: Additional file 7: Table S2 — Codon Usage Bias Table. [file 1471-2180-12-188-S7.pdf]

Gene	ENC	CBI	SChi^2	GC3	GC
***P. syringae* pv phaseolicola** GC=58.76%, GC3=70.05%*					
Hrc1 T3SS					
hrcV	42.023	0.479	0.384	0.746	0.591
hrcC	46.292	0.393	0.285	0.680	0.580
hrcN	41.804	0.507	0.365	0.733	0.639
$hrcQ_A$	41.407	0.500	0.454	0.734	0.658
$hrcQ_B$	43.626	0.492	0.490	0.688	0.623
hrpK	46.163	0.369	0.212	0.687	0.591
hrpQ	42.712	0.446	0.462	0.692	0.604
2nd T3SS					
$hrc_{II}V$	44.812	0.437	0.427	0.678	0.599
$hrc_{II}C_1$	48.114	0.440	0.338	0.689	0.609
$hrc_{II}C_2$	42.594	0.480	0.377	0.721	0.608
$hrc_{II}N$	48.278	0.413	0.262	0.684	0.628
$hrc_{II}Q$	48.700	0.422	0.315	0.693	0.617
$hrp_{II}K$	46.326	0.391	0.251	0.691	0.566
$hrp_{II}Q$	53.515	0.357	0.257	0.611	0.591
***P. syringae* pv tabaci** GC=57.80%, GC3=68.61%*					
2nd T3SS					
$hrc_{II}V\_N$	44.798	0.462	0.400	0.699	0.601
$hrc_{II}V\_C$	46.157	0.441	0.506	0.611	0.595
$hrc_{II}C_1$	53.571	0.410	0.297	0.653	0.599
$hrc_{II}C_2\_N$	50.221	0.402	0.270	0.700	0.611
$hrc_{II}C_2\_C$	36.481	0.613	0.851	0.722	0.601
$hrc_{II}N\_N$	48.976	0.456	0.303	0.727	0.632
$hrc_{II}N\_C$	45.027	0.497	0.515	0.665	0.625
$hrc_{II}Q$	50.607	0.403	0.292	0.674	0.608
$hrp_{II}Q$	54.875	0.328	0.240	0.599	0.588
$hrp_{II}K$	46.469	0.379	0.251	0.684	0.566
***Rhizobium* sp. NGR234** GC=62.35%, GC3=76.55%*					
Rhc_I T3SS					
rhcV	54.210	0.280	0.144 (0,119)	0.631	0.751
$rhcC_1$	54.856	0.367	0.239 (0.148)	0.634	0.559
$rhcC_2$	56.318	0.286	0.183 (0.143)	0.610	0.561
rhcN	54.479	0.321	0.154 (1.113)	0.631	0.608
rhcQ	58.482	0.281	0.169 (0.122)	0.605	0.582
hrpQ-like	54.985	0.314	0.219 (0.145)	0.569	0.552
Rhc_{II}T3SS					
$rhc_{II}V$	39.213	0.558	0.318 (0.277)	0.819	0.655
$rhc_{II}C_1$	45.007	0.486	0.316 (0.208)	0.771	0.624

$rhc_{II}C_2$	36.204	0.652	0.450 (0.374)	0.859	0.646
$rhc_{II}N$	36.611	0.594	0.382 (0.314)	0.680	0.679
$hrp_{II}Q$	40.613	0.555	0.284 (0.195)	0.831	0.671
$rhc_{II}Q$	42.736	0.509	0.274 (0.198)	0.784	0.652
B. japonicum GC=65.59%, GC3=86.46%*					
rhcV	48.594	0.329	0.209	0.646	0.570
$rhcC_1$	55.937	0.303	0.192	0.642	0.590
$rhcC_2$	47.759	0.370	0.316	0.614	0.603
rhcN	45.896	0.415	0.244	0.671	0.619
rhcQ	50.065	0.364	0.221	0.666	0.620
y4yQ	47.525	0.383	0.313	0.622	0.582
***R. etli* CNF 42** GC=61.96%, GC3=76.72%*					
rhcV	39.671	0.562	0.379	0.818	0.647
$rhcC_1$	37.070	0.637	0.663	0.819	0.645
rhcN	37.576	0.575	0.376	0.817	0.667
rhcQ	42.110	0.533	0.359	0.773	0.633
$hrpK_1$	38.348	0.541	0.402	0.794	0.619
$hrpK_2$	38.047	0.527	0.397	0.808	0.608

Additional file 7. Table S2 : Codon Usage Bias Table.

SChi2: Scaled Chi-square, (Scaled Chi-square computed without Yates' correction)

ENC: Effective number of codons,

CBI: Codon Bias Index,

GC3: G+C content at (synonymous) third codon positions,

*source: www.kazusa.or.jp

SChi2, Scaled Chi Square (Shields et al. 1988)

The "scaled" 2 (chi square) is a measure based on the chi square statistics; i.e., based on the difference between the observed number of codons and those expected from equal usage of codons. The sum of the chi square values is divided by the total number of codons in the gene excluding those codons coding for a unique amino acid;

i.e. all codons excluding the Trp and Met codons (nuclear universal genetic code). DnaSP can compute the "scaled" chi square with Yates' correction, and also assuming a given G+C content (by default the G+C content is 50%).

CBI, Codon Bias Index (Morton 1993)

CBI is a measure of the deviation from the equal use of synonymous codons. CBI values range from 0 (uniform use of synonymous codons) to 1 (maximum codon bias).

ENC, Effective Number of Codons (Wright 1990)

That measure quantifies the "effective" number of codons that are used in a gene. For the nuclear universal genetic code, the value of ENC ranges from 20 (only one codon is used for each amino acid; i.e., the codon bias is maximum) to 61 (all synonymous codons for each amino acid are equally used; i.e., no codon bias).

Yates, F. (1934). "Contingency table involving small numbers and the χ^2 test". *Supplement to the Journal of the Royal Statistical Society* **1**(2): 217–235.

Shields, D.C. et al. (1988). "Silent" sites in Drosophila genes are not neutral: Evidence of selection among synonymous codons. *Mol.Bio. Evol.* 5: 704-716.

Morton, B.R. (1993). Chloroplast DNA codon use: Evidence for selection at the psb A locus based on tRNA availability. J. Mol. Evol. 37: 273-280.

Wright, F. (1990). The "effective number of codons" used in a gene. *Gene* 87: 23-29
